# Terrestrial Mollusca of Cuc Phuong National Park, Vietnam – Results from the 2019 VIETBIO inventory work

**DOI:** 10.3897/BDJ.13.e163277

**Published:** 2025-09-30

**Authors:** Parm Viktor von Oheimb, Anna Sulikowska-Drozd, Thuy Dieu Dinh, Nora Lentge-Maaß, Tu Van Do, Katharina C. M. von Oheimb

**Affiliations:** 1 Museum für Naturkunde – Leibniz Institute for Evolution and Biodiversity Science, Berlin, Germany Museum für Naturkunde – Leibniz Institute for Evolution and Biodiversity Science Berlin Germany; 2 Department of Invertebrate Zoology and Hydrobiology, Faculty of Biology and Environmental Protection, University of Łódź, Łódź, Poland Department of Invertebrate Zoology and Hydrobiology, Faculty of Biology and Environmental Protection, University of Łódź Łódź Poland; 3 Vietnam National Museum of Nature, Vietnam Academy of Science and Technology, Ha Noi, Vietnam Vietnam National Museum of Nature, Vietnam Academy of Science and Technology Ha Noi Vietnam; 4 Graduate University of Science and Technology, Vietnam Academy of Science and Technology, Ha Noi, Vietnam Graduate University of Science and Technology, Vietnam Academy of Science and Technology Ha Noi Vietnam; 5 Institute of Ecology and Biological Resources, Vietnam Academy of Science and Technology, Ha Noi, Vietnam Institute of Ecology and Biological Resources, Vietnam Academy of Science and Technology Ha Noi Vietnam

**Keywords:** biodiversity discovery, Gastropoda, protected area, scientific collection, Southeast Asia, taxonomic inventory

## Abstract

**Background:**

Cuc Phuong National Park is located along a range of limestone karst mountains in northern Vietnam and spans over an area of 222 km² mainly covered by tropical evergreen forest. While a number of works have focused on the park’s rich terrestrial mollusc fauna to date, the most extensive survey so far was carried out in 1998. In the present paper with its corresponding data package, we focus on the land snails and slugs recorded in the park during the VIETBIO inventory work between 29 April and 10 May 2019. Throughout this survey, live specimens and empty shells of terrestrial molluscs were collected at 34 sampling sites via visual search and additional soil sampling. Furthermore, we summarise the current knowledge on the park’s land molluscs.

**New information:**

We present new data on the terrestrial mollusc fauna of Cuc Phuong National Park, which are linked to collection material stored at the Museum für Naturkunde Berlin (dry shells, wet specimens, samples for long-term tissue preservation) and to a smaller part at the Institute of Ecology and Biological Resources, Ha Noi (dry shells). Our data package contains a Darwin Core Archive with respective collection data, and four further data sets with photos of sampling sites and live specimens. We provide soil pH values and information on the microhabitats where live specimens were collected. In addition, the paper includes photos of collection material, which were partly taken with a DORA station developed for the high-volume imaging of molluscan specimens. In total, 116 species and 1 additional subspecies of land snails and slugs from 23 families were recorded. From the (sub-)species found, we matched 70 with nominal species-group taxa, while 47 remained provisionally named, with most of the latter likely belonging to undescribed species. However, as the taxonomic identification was only based on shell morphology, external features of the soft body, and sampling locality, it should still be regarded as provisional. We collected a total of 2666 specimens, 1909 of them alive and 757 as empty shells. From all taxa recorded, ca. 26% were only found at one sampling site each and ca. 15% were represented only by a single individual, which indicates that many species are rare or unevenly distributed. Based on our survey and previous works, we compiled a comprehensive list of 159 species and 1 additional subspecies known from Cuc Phuong National Park, about one-third of them non-eupulmonates, which places the park amongst the most species-rich tropical forest regions known worldwide. A mark-recapture analysis, based on the 1998 collection and our survey, resulted in an estimate of 178 ± 7 species of shelled land molluscs present in the park. When adding further (semi-)slug species that were disregarded for this analysis, at least 184 ± 7 terrestrial mollusc species can be expected. The high overall number of species in the park probably results from a combined effect of allopatric, mosaic, and sympatric diversity.

## Introduction

Since the mid-20^th^ century, various protected areas have been established across Vietnam in order to preserve the country’s rich and unique biodiversity (Sterling et al. 2006, Chu et al. 2020). Cuc Phuong National Park, which is located at the southern edge of the Red River Delta in northern Vietnam (Duwe et al. 2022), was declared a protected forest in 1962 and became Vietnam’s first national park in 1966 (Chu et al. 2020). The park is situated along a range of limestone karst mountains and spans over an area of 222 km² that is mainly covered by broad-leaved tropical evergreen lowland forest (see Duwe et al. 2022).

Limestone karst environments in Vietnam provide habitat for various organisms with high calcium requirements and they hold a rich diversity and abundance of terrestrial molluscs (Clements et al. 2006, Raheem et al. 2017). For calcium-dependent mollusc species, surrounding non-limestone regions can act as dispersal barriers (Tweedie 1961, Schilthuizen 2004, Clements et al. 2008) and such taxa often have relatively small distribution ranges, while species communities can differ from one karst area to another (Tweedie 1961, Tongkerd et al. 2004, Clements et al. 2006, von Oheimb et al. 2018, Sutcharit et al. 2020). Overall, knowledge about the diversity and distribution of Vietnam’s terrestrial mollusc species is still limited (Raheem et al. 2017) and even protected areas such as Cuc Phuong National Park are far from being comprehensively studied.

The present paper with its corresponding data package focuses on the terrestrial Mollusca recorded in Cuc Phuong National Park during the 2019 VIETBIO inventory work, which aimed to survey the biodiversity of various organism groups within the park and to obtain samples for further research (Duwe et al. 2022, Mey and Vu 2022, Mey et al. 2023, Hayden Bofill et al. 2024). The field work was carried out under the framework of the bilateral German-Vietnamese project “Innovative Approaches to Biodiversity Discovery and Characterisation in Vietnam” (VIETBIO); for the general approach and methodology used during the 2019 VIETBIO field trip, and an overview on Cuc Phuong National Park and its biodiversity, see the editorial paper of Duwe et al. (2022).

The so far most extensive survey of the park’s terrestrial mollusc fauna was conducted in 1998. It was part of a land and freshwater mollusc assessment in several regions of northern Vietnam, performed by JJ Vermeulen and AJ Whitten. The results from Cuc Phuong National Park were included in a subsequently released report (Vermeulen and Whitten 1998) and later updated for another, more detailed report with a more thorough species identification (Vermeulen and Maassen 2003). The sampling was performed at four different localities within Cuc Phuong National Park and focused on the collection of soil samples, while larger mollusc specimens were collected by hand (Vermeulen and Whitten 1998, Vermeulen and Maassen 2003). The 1998 collection was later split into three sets, one was deposited in Vietnam and two were retained by JJ Vermeulen and WJM Maassen (Vermeulen and Maassen 2003). While the set in Vietnam unfortunately has not been preserved, we were able to use material from the set of JJ Vermeulen for comparison. In total, 124 terrestrial gastropod species have been identified from the 1998 collection by Vermeulen and Maassen (2003) (three additional species of (semi-)slugs were only mentioned in Vermeulen and Whitten (1998)), many of them presumably undescribed and assigned with provisional codes instead of species names.

Beside the 1998 survey, a small-scale study focusing only on operculate land snails was carried out in 2005 resulting in a total number of 10 recorded species (Yamazaki et al. 2007). The 2010 management report for Cuc Phuong National Park (Nguyen and Nguyen 2010) mentioned 129 gastropod species from 24 families for the park without providing references or a list of the individual species. Furthermore, several studies on particular terrestrial gastropod taxa from Cuc Phuong National Park, including descriptions of new species, have been published (see Table 1 for a list of nominal species-group taxa with a type locality in Cuc Phuong National Park and respective references).

In this data paper, we present a package of terrestrial mollusc collection data from Cuc Phuong National Park, which are linked to preserved material housed at the Museum für Naturkunde Berlin and the Institute of Ecology and Biological Resources, Ha Noi. Amongst these data, we provide photos of sampling sites and live animals, soil pH values and information on microhabitats. In addition, this paper includes a selection of photos that result from the digitisation of preserved specimens at the Museum für Naturkunde Berlin. Furthermore, we summarise the knowledge on the park’s terrestrial malacofauna, provide a comprehensive species list and present an estimation for the total species richness of shelled land molluscs in the park, based on the 1998 collection and our sampling utilising a mark-recapture approach.

## Sampling methods

### Sampling description

The field work in Cuc Phuong National Park, Vietnam, was carried out to collect specimens from all groups of terrestrial Mollusca and aimed to record as many different species as possible. Sampling took place from 29 April to 10 May 2019 and was carried out by PV von Oheimb, KCM von Oheimb, A Sulikowska-Drozd and TD Dinh, supported by TV Do and other members of the VIETBIO team (Table 2, data package; see Duwe et al. 2022 for a list of all 2019 VIETBIO field trip participants).

At sampling sites (sampling points or sampling transects), live specimens and empty shells of terrestrial gastropods were detected via visual search, which is a standard method for conducting species inventory in this group (Cameron and Pokryszko 2005). Specimens were collected by hand, partly using spring steel tweezers (and, on one occasion, using an insect hand net). Live specimens were kept alive for further processing, with the exception of minute individuals, which were directly transferred into 1.5 ml tubes filled with 75% denatured ethanol. In addition, soil samples were taken at selected sampling sites for improving the chances of finding small species of terrestrial molluscs (Cameron and Pokryszko 2005). To obtain the soil samples, which had a total volume of >1 litre, small amounts (approx. a handful) of leaf litter and soil from the upper layer were collected from different spots within one sampling site and partly sieved on site with a 5 mm mesh size sieve (for further processing of soil samples, see below).

For most of the material that was collected alive, we recorded the general microhabitat where each specimen was found. Microhabitats were classified as vegetation (living plants, upright tree-trunks), rock (rocky surface), ground (leaf litter and other materials on the ground, soil surface) and soil (extracted from soil samples). For species well distinguishable by the naked eye during visual search, we omitted further sampling of live specimens and shells from the respective species at a given site, as soon as several preferably live specimens were collected. We also omitted to collect juvenile specimens when a sufficient number of adult specimens of the respective species could be collected at a site. Both was regularly the case, as live gastropods and empty shells were often present in relatively high abundances.

Primary sampling data, including GPS-based geographic coordinates (Table 2, data package) and sampling site photos (data package), were recorded in the field using the prototypic mobile app MyFieldBook (see Duwe et al. 2022). The elevation data given in Table 2 were inferred retrospectively based on the Amazon Web Services Terrain Tiles (https://registry.opendata.aws/terrain-tiles, retrieved on 23 May 2024) using the package elevatr version 0.99.0 (Hollister et al. 2023) in R version 4.4.0 (R Core Team 2024) and rounded to whole metres above sea level.

### Step description

Some of the specimens collected were photographed alive, partly at the respective sampling site and mostly *ex situ*. Live gastropods collected by visual search were relaxed by drowning them at room temperature in re-sealable plastic bags filled with tap water and added menthol crystals (Pearce and Örstan 2006). After relaxation, these specimens were transferred in 75% denatured ethanol. The collected soil samples were dried and sieved with different mesh sizes (5 mm, 2 mm, 1 mm). All sieve fractions were carefully examined under a stereomicroscope, while live gastropods and empty shells were extracted using spring steel tweezers. Live specimens from the soil samples were directly transferred into 75% denatured ethanol. The ethanol from all wet specimens was exchanged after a few days.

Soil pH was measured from each soil sample following Anderson and Ingram (1993). For this, 20 g (± 0.03 g) of soil from the 2 mm sieve fraction (partly after careful pestling) was mixed with 50 ml deionised water. The mixture was stirred for 10 min (at 140 rpm), left to stand for 30 min and stirred again for 2 min, before the pH of the supernatant liquid was measured with a 766 Laboratory pH Meter (Knick Elektronische Messgeräte GmbH & Co. KG, Berlin, Germany).

Badly damaged and heavily worn empty shells were discarded, except in cases where these were the only samples of the respective species from that site. A number of individuals (only Clausiliidae) were kept alive in laboratory culture at the University of Lodz for some time and were preserved later (see data package, Suppl. material 1). In some cases, dry shells were obtained from specimens collected alive. For this, respective ethanol-preserved material was either dried entirely, dried after (partial) soft body removal, or dried after boiling and (partial) soft body removal. In case of operculate gastropods, the operculum was kept.

A total of 116 ethanol-preserved specimens belonging to 69 different (sub-)species were selected for long-term tissue preservation. One or several pieces of soft body (preferably foot tissue) from these specimens or, in case of small species, the complete specimens were transferred into 96% ethanol and stored frozen at the Museum für Naturkunde Berlin (data package, Suppl. material 1). To avoid contamination during tissue preparation, all dissecting tools used were placed in a ca. 0.7% sodium hypochlorite solution (mixture of deionised water and household bleach) for several minutes and subsequently rinsed twice with deionised water between the preparation of different specimens.

The main part of the collection is stored in Germany, at the facilities of the Museum für Naturkunde Berlin (dry shells, wet specimens and samples for long-term tissue preservation; catalogue number prefix ZMB), and a smaller part in Vietnam, at the Institute of Ecology and Biological Resources, Ha Noi (dry shells; catalogue number prefix IEBR) (data package, Suppl. material 1).

**Figure 1.**
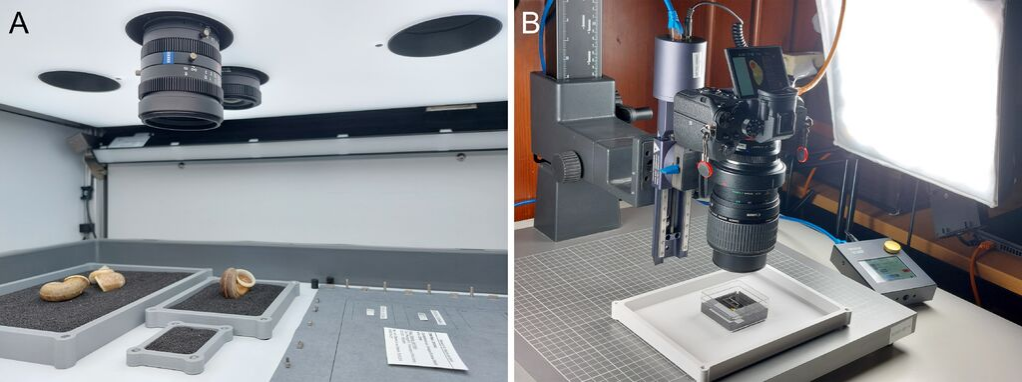
Digitisation stations at the Museum für Naturkunde Berlin used for photographing terrestrial Mollusca specimens collected during the 2019 VIETBIO inventory work in Cuc Phuong National Park. **A** View inside the box-shaped main device of the DORA station. Dry shells are placed in sand-filled inserts. Three cameras for taking photos of specimens and another one for labels are installed above. The top light is activated. **B** Macro photography station. Wet material is placed on sand in ethanol. The camera is mounted on a focusing rack and the lateral lights are activated.

A fraction of the material stored at the Museum für Naturkunde Berlin was photographed according to the digitisation standards that have been developed for the museum’s Mollusca collection, based on the recommendations of Callomon (2019). We aimed to digitise at least one specimen of each (sub-)species, while uncertainly identified, juvenile and damaged material was so far mostly omitted. Two digitisation stations were used, a DORA station (Fig. 1A), mainly for specimens with a maximum dimension >5 mm, and a macro photography station (Fig. 1B), mainly for smaller specimens. The DORA station has been developed for the high-volume imaging of molluscan specimens between 2020 and 2022 by a team of digitisation experts from the Museum für Naturkunde Berlin, including N Lentge-Maaß, and from the Fraunhofer Institute for Factory Operation and Automation (IFF), Magdeburg. Inside the box-shaped main device of the DORA station, three Manta G-1236 industrial cameras (Allied Vision, Stadtroda, Germany) with different Zeiss Dimension lenses (50 mm, 25 mm and 18 mm focal length; Carl Zeiss AG, Oberkochen, Germany) are installed. Lights at the top, front and back of the device’s inside can be set to different lighting scenarios. An additional one-shot camera is installed for digitising label information. The DORA station was operated with the DORA-Aquisition 1.4 software (Fraunhofer IFF, Magdeburg, Germany). For the digitisation of specimens, they were placed on the device’s sliding drawer in one of three differently-sized inserts filled with quartz sand, each insert below one of the main cameras. Dry shells were usually placed directly in the inserts and wet specimens in additional, sand-filled plastic boxes with ethanol. The cameras then took multiple photos at different focal distances, which were stacked with the Helicon Focus Pro 8.2.2 software (Helicon Soft, Kharkiv, Ukraine). QR-codes on labels were automatically scanned, and a colour chart and a scale bar were included in each photo. Final images were stored as DNG and PNG files. For each image, a JSON file containing the respective metadata was created. JSON files originating from the same lot were later combined into a single file. The macro photography station included a copy stand with a camera mount, a Sony α7R III camera (Sony Group Corporation, Tokyo, Japan) with different macro lenses, and two lateral lights. Specimens placed on quartz sand, either dry or in ethanol, were photographed with a grey card (Novoflex, Memmingen, Germany) and a scale. A Castel-Micro focusing rack (Novoflex, Memmingen, Germany) was utilised to take photos at different focal distances and the Capture One 20 Pro 13.1.3.13 software (Capture One, Frederiksberg, Denmark) was used for image capture. Photos were stacked with the Helicon Focus Pro 8.2.2 software. The final specimen images were stored as DNG and TIF files, and respective metadata were compiled in JSON files. Photos taken with both digitisation stations and the corresponding metadata will be made available via the data portal of the Museum für Naturkunde Berlin, while a selection is included in the present paper (see results).

The identification of (sub-)species was based on shell morphology, external features of the soft body, and sampling locality. No DNA sequence data, dissections and studies of the radula have been taken into account, and the identification and species delimitation should still be regarded as provisional, in particular for the provisionally-named morphospecies (see below). Besides literature that provided general orientation (Schileyko 2011, Raheem et al. 2017, Páll-Gergely et al. 2020a), we aimed to take all relevant taxonomic works for the region into account (see Table 3 for literature used to identify the individual taxa). We compared our material with descriptions and figures from the literature and with photos of type specimens. In addition, we compared our material with parts of JJ Vermeulen’s set of the 1998 collection, which is today stored in his personal collection in Leiden and at the Naturalis Biodiversity Center, Leiden. The material available to us contained dry shells of most species listed for Cuc Phuong National Park by Vermeulen and Maassen (2003) (see Table 3). Besides this material, we also utilised few additional non-Cuc Phuong National Park samples studied by Vermeulen and Maassen (2003), all from the personal collection of JJ Vermeulen, as well as unpublished photos and drawings of shells provided by him, which covered some, but not all, species absent from the Cuc Phuong National Park material available to us (see Table 3; no material or images of the (semi-)slugs only mentioned in Vermeulen and Whitten (1998) were available to us). Furthermore, we used material from the collection of the Museum für Naturkunde Berlin, including type and topotypical material, for comparison. The identification of *Amphidromus roseolabiatus* Fulton, 1896 followed the study of Do et al. (2024), which included material from our 2019 collection.

We generally followed the identification of Vermeulen and Maassen (2003) in respect of species listed by them for Cuc Phuong National Park, except for cases where we disagreed with their identification or species delimitation, or where more recent literature allowed an improved identification. In cases where taxa did not match any described species or could not be identified without further study, we used provisional codes for distinguishing morphospecies. These codes were adopted from Vermeulen and Maassen (2003), and Jirapatrasilp et al. (2021) (genus *Quantula*), or were newly assigned (sp. A, sp. B, etc.). For two species of the genus *Cyclophorus*, we adopted the names of the respective lineages from von Oheimb et al. (2018). In cases where we placed a species, which was assigned with a provisional code by Vermeulen and Maassen (2003), in a different genus, we did not apply the same code (for example, *Trochomorpha* sp. A corresponds to *Videna* sp. vi-1 *sensu* Vermeulen & Maassen 2003; see Table 4). Based on the material available to us, Vermeulen and Maassen (2003) had lumped what we regarded as two morphospecies under the single provisional code *Microcystina* sp. vi-b04; in this case, we applied two new codes, *Microcystina* sp. B and *Microcystina* sp. E, for the respective species for the reason of clarity (see Table 4).

The life stage “juvenile” indicates samples that consist exclusively of specimens that were clearly identified as juvenile (data package, Suppl. material 1). We aimed to identify juveniles by comparison to adults; nevertheless, the taxonomic identification of juveniles was often difficult and is not as reliable as for adult material. Samples that consist exclusively of specimens with uncertain identification (such as samples of small juveniles or damaged specimens) are marked with the uncertainty indicator “cf.” (data package, Suppl. material 1).

The suprageneric classification follows Bouchet et al. (2017), with the exception of Pollicariidae and Hypselostomatidae, which are treated as families in accordance with recent studies (Páll-Gergely et al. 2023, Gojšina et al. 2025, Jirapatrasilp et al. 2025), and of Architaenioglossa and Littorinimorpha, which are regarded as orders following MolluscaBase eds. (2025). For species-group names established by TN Nguyen, we cite the author’s given name “Thach” as practised by him and other authors (Nguyen 2018, Páll-Gergely et al. 2020a); elsewhere this author is cited with his family name for consistency.

Due to the nested structure of the Mollusca collection at the Museum für Naturkunde Berlin, the Darwin Core Archive of the data package includes occurrence data on both, specimen lots and specimens that were separated within such lots. For convenience, we provide Suppl. material 1, an additional tab-delimited CSV file with each specimen represented only once, including all relevant occurrence data from the Darwin Core Archive (and few additional data on life stage and identification uncertainty for remaining material from lots where specimens were separated).

Based on the taxonomic identification of the collected material, the comparison with the 1998 collection, and plausible reports from the literature, a list of all terrestrial Mollusca reported from Cuc Phuong National Park was compiled. No collection material of taxa that were not found in 2019 was evaluated for this purpose. When appropriate and possible, species names have been updated, based on the literature.

Mark-recapture procedures, which are used to estimate the size of animal populations by marking captured individuals and evaluating the proportion of marked individuals in a subsequent recapture, can also be used for estimating the species number of a particular group within an area (see Burnham and Overton 1979, Boulinier et al. 1998). Such practice is grounded in an analogy between captured individuals and recorded species. We utilised a mark-recapture approach for estimating the total species richness of shelled land molluscs in Cuc Phuong National Park, based on the species recorded from the 1998 collection (according to Vermeulen and Maassen (2003) and an evaluation of material from that collection available to us), as well as on those recorded from the 2019 collection. The analysis was conducted with the programme SPECRICH2 in the software package COMDYN (Hines et al. 1999). SPECRICH2 is based on the *M*_h_ model with the associated jackknife estimator, which accounts for heterogeneity amongst species in detection probability (Burnham and Overton 1979). For the analysis, we considered 124 species recorded in 1998 (n_1_), 110 species recorded in 2019 (n_2_), 62 species recorded from one collection (f_1_) and 86 species recorded from both collections (f_2_). Full details on the data used for the analysis are given in Table 4. For standard error computation, we used the default number of 200 iterations for the bootstrap estimator and the default number seed. The standard error was rounded to the nearest integer. As they were not included in the species list of Vermeulen and Maassen (2003), all species of slugs (belonging to the families Rathouisiidae, Veronicellidae and Philomycidae) and the ariophantid semi-slug genus *Parmarion* recorded in 2019 were disregarded for the analysis. Species, for which only uncertainly identified material was available in the 2019 collection (see Table 4), were nevertheless included as they were clearly heterospecific from the other species in that collection. As the analysis focused on species richness, data from both subspecies of *Megalauchenia proctostoma* were combined. In cases of conflict in species delimitation, we always followed the systematics used in the present study (see Table 4 for details). *Trochomorpha saigonensis* was regarded as present in the 1998 collection because one specimen was present in an examined 1998 sample of *Trochomorpha* sp. A. *Oospira abstrusa*, *Oospira eregia* and *Durgella* sp. B were regarded as present in the 1998 collection, based on a presumed concordance with species in Vermeulen and Maassen (2003), from which no material could be examined (see Table 4).

## Geographic coverage

### Description

**Figure 2.**
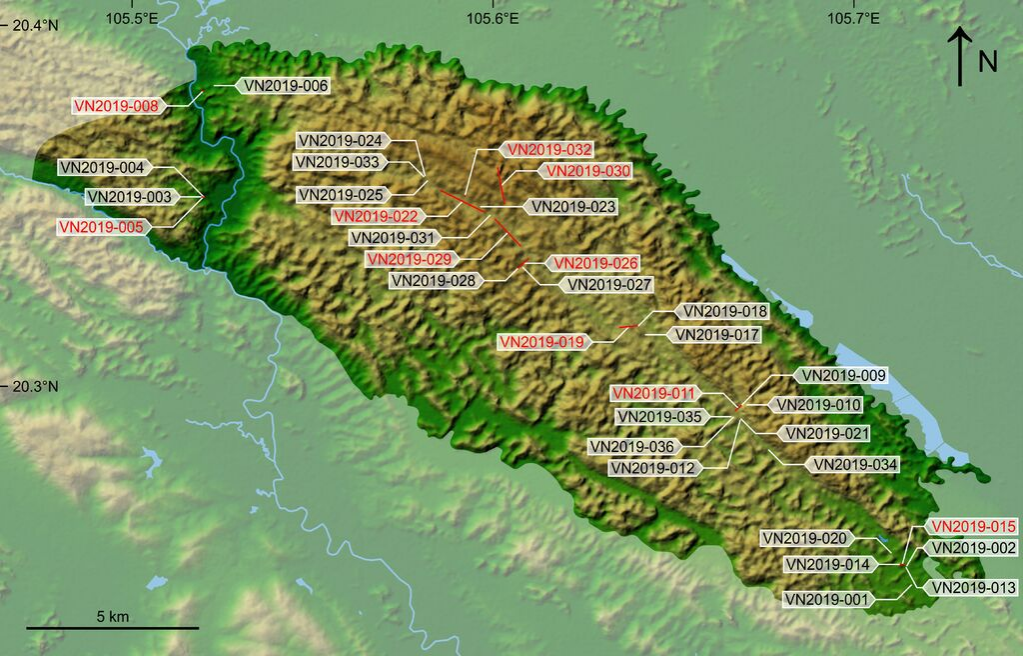
Terrain map of Cuc Phuong National Park (highlighted) with sampling sites of terrestrial Mollusca from the 2019 VIETBIO inventory work. The eventID is in black for single sampling points and in red for sampling transects. The starting and end points of each sampling transect are indicated by a connecting red line. The map was prepared in QGIS 3.34.14 (QGIS Association, Laax, Switzerland), based on the Shuttle Radar Topography Mission (SRTM) data set, while data on waterbodies and the outline of Cuc Phuong National Park were derived from OpenStreetMap contributors (https://www.openstreetmap.org/copyright, retrieved on 7 March 2025).

Specimen collection was carried out at 34 sampling sites within Cuc Phuong National Park, Vietnam (Fig. 2, Table 2, data package). Of these, 24 represented single sampling points, where the collection of specimens took place within a ca. 50 m diameter circle. The other 10 represented sampling transects with a starting and end point.

## Usage licence

### Usage licence

Other

### IP rights notes

Creative Commons Attribution License (CC BY 4.0)

## Data resources

### Data package title

Terrestrial Mollusca of Cuc Phuong National Park, Vietnam – Results from the 2019 VIETBIO inventory work. Data package

### Number of data sets

5

### Data set 1.

#### Data set name

Terrestrial Mollusca of Cuc Phuong National Park, Vietnam – Results from the 2019 VIETBIO inventory work. Collection data

#### Data format

Darwin Core Archive

#### Character set

UTF-8

#### Download URL

https://doi.org/10.7479/4ers-3029

#### Description

Collection data of terrestrial Mollusca from the 2019 VIETBIO inventory work in Cuc Phuong National Park, Vietnam. Version 1.0

### Data set 2.

#### Data set name

Terrestrial Mollusca of Cuc Phuong National Park, Vietnam – Results from the 2019 VIETBIO inventory work. Photos 1/4: sampling sites

#### Data format

JPG files

#### Download URL

https://doi.org/10.7479/vnrr-em57/1

#### Description

Photos of sampling sites of terrestrial Mollusca from the 2019 VIETBIO inventory work in Cuc Phuong National Park, Vietnam. Version v1

### Data set 3.

#### Data set name

Terrestrial Mollusca of Cuc Phuong National Park, Vietnam – Results from the 2019 VIETBIO inventory work. Photos 2/4: live non-stylommatophoran gastropods

#### Data format

JPG files

#### Download URL

https://doi.org/10.7479/vnrr-em57/2

#### Description

Photos of live non-stylommatophoran gastropods from the 2019 VIETBIO inventory work in Cuc Phuong National Park, Vietnam. Version v1

### Data set 4.

#### Data set name

Terrestrial Mollusca of Cuc Phuong National Park, Vietnam – Results from the 2019 VIETBIO inventory work. Photos 3/4: live non-camaenid stylommatophoran gastropods

#### Data format

JPG files

#### Download URL

https://doi.org/10.7479/vnrr-em57/3

#### Description

Photos of live non-camaenid stylommatophoran gastropods from the 2019 VIETBIO inventory work in Cuc Phuong National Park, Vietnam. Version v1

### Data set 5.

#### Data set name

Terrestrial Mollusca of Cuc Phuong National Park, Vietnam – Results from the 2019 VIETBIO inventory work. Photos 4/4: live camaenid gastropods

#### Data format

JPG files

#### Download URL

https://doi.org/10.7479/vnrr-em57/4

#### Description

Photos of live camaenid gastropods from the 2019 VIETBIO inventory work in Cuc Phuong National Park, Vietnam. Version v1

## Additional information

### Results

**Figure 3.**
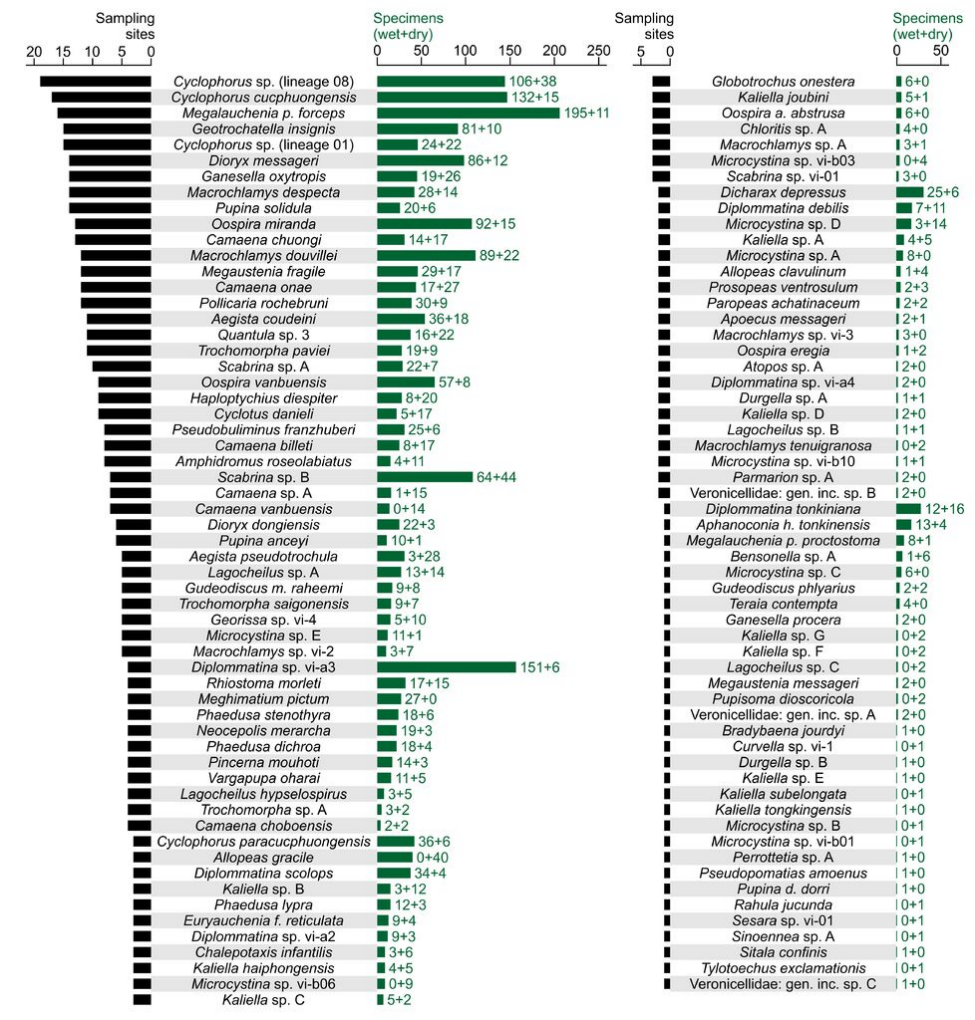
Bar chart showing the number of sampling sites (black) and of collection specimens (green) per (sub-)species recorded during the 2019 VIETBIO inventory work in Cuc Phuong National Park. Next to bars, respective numbers of wet (first summand) and dry (second summand) collection specimens are given.

During the field work in Cuc Phuong National Park, 2666 specimens of terrestrial Mollusca were collected, 1909 of them alive (40 of those were later used to obtain dry shells) and 757 as empty shells (data package, Suppl. material 1). In total, 116 species and 1 additional subspecies of terrestrial molluscs were recorded, which belong to 23 families (Table 3). From all (sub-)species recorded, 70 were matched with nominal species-group taxa, while 47 remained provisionally named (Table 3). The families with the highest number of species found were Ariophantidae (18 species), Cyclophoridae (17 species) and Camaenidae (16 species) (Table 3). Most material was collected from representatives of the families Cyclophoridae (783 specimens), Clausiliidae (470 specimens) and Camaenidae (345 specimens) (data package, Suppl. material 1). From the 1623 live specimens for which the microhabitat was recorded, 589 were collected on the vegetation (58 species and 1 additional subspecies), 514 on rock (52 species and 1 additional subspecies), 324 on the ground (40 species) and 196 from the soil (23 species) (data package, Suppl. material 1). The number of sampling sites, from which specimens of a (sub-)species were collected, ranged from 19 to 1 (Fig. 3). The number of collected specimens per (sub-)species was between 206 and 1 (Fig. 3). From the 117 (sub-)species found, 31 were only recorded from one sampling site each (ca. 26%) and 17 only represented by a single specimen (ca. 15%).

Across the seven sampling sites where soil samples were taken, pH values ranged from 6.78 to 7.68 (Table 2, data package), thus falling in the neutral (6.6 to 7.3) and slightly alkaline (7.4 to 7.8) range (Soil Science Division Staff 2017). The average soil pH was 7.18.

**Figure 4.**
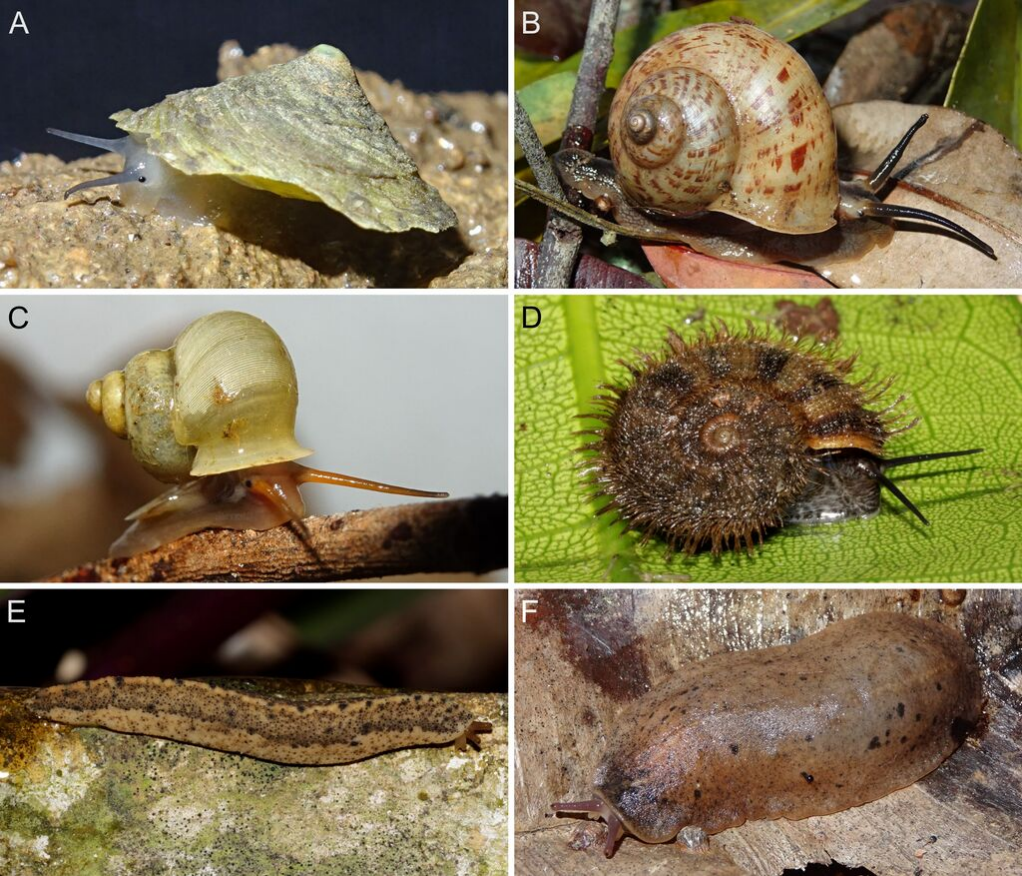
Photos of live gastropods from Cuc Phuong National Park. Specimens were collected in 2019 and are stored at the Museum für Naturkunde Berlin. **A** *Geotrochatella insignis* Dautzenberg, 1896; ZMB.Moll 291144-1. **B** *Cyclophorus paracucphuongensis* Oheimb, 2019; ZMB.Moll 291035a-2. **C** *Dioryx messageri* (Bavay & Dautzenberg, 1900); ZMB.Moll 290156a-1. **D** *Scabrina* sp. vi-01 *sensu* Vermeulen & Maassen 2003; ZMB.Moll 291114. **E** *Atopos* sp. A; ZMB.Moll 266078. **F** Veronicellidae: gen. inc. sp. B; ZMB.Moll 265817.

**Figure 5.**
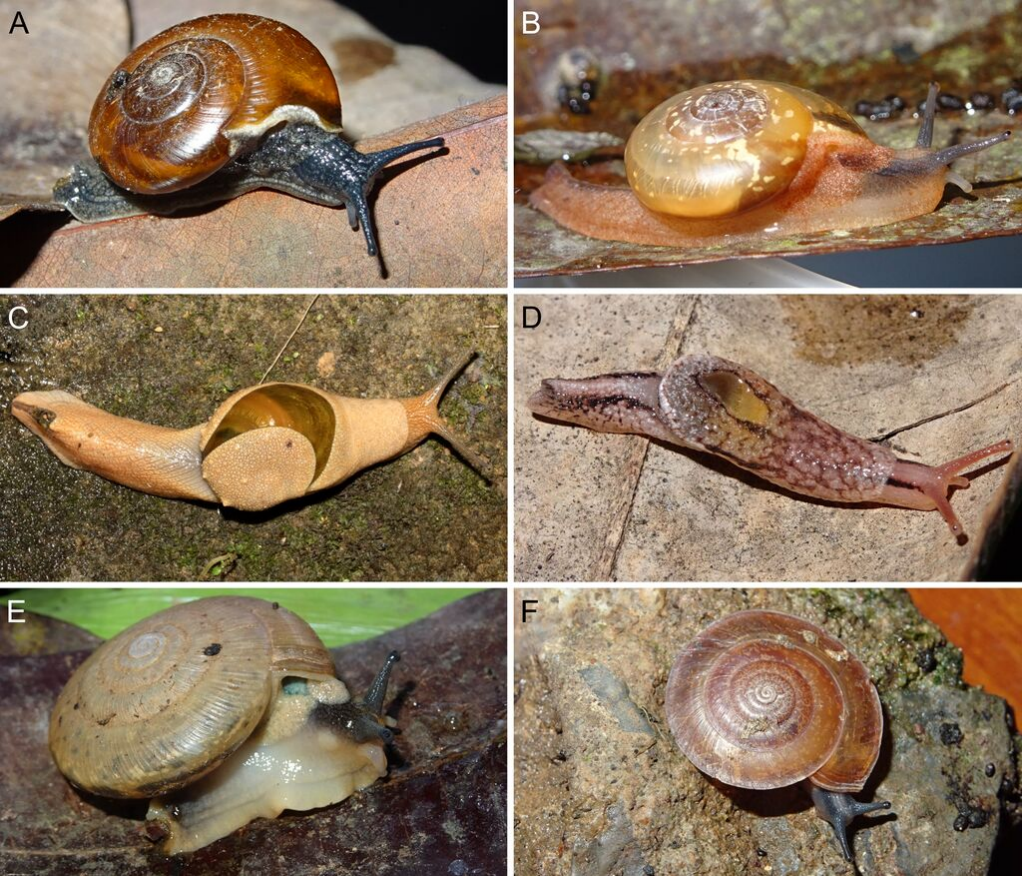
Photos of live gastropods from Cuc Phuong National Park. Specimens were collected in 2019 and are stored at the Museum für Naturkunde Berlin. **A** *Macrochlamys douvillei* Dautzenberg & Fischer, 1906; ZMB.Moll 290203. **B** *Macrochlamys* sp. vi-3 *sensu* Vermeulen & Maassen 2003; ZMB.Moll 290184. **C** *Megaustenia messageri* (Bavay & Dautzenberg, 1909); ZMB.Moll 250152. **D** *Parmarion* sp. A; ZMB.Moll 290252. **E** *Quantula* sp. 3 *sensu* Jirapatrasilp et al. 2021; ZMB.Moll 290173-1. **F** *Trochomorpha paviei* (Morlet, 1885); ZMB.Moll 291222.

**Figure 6.**
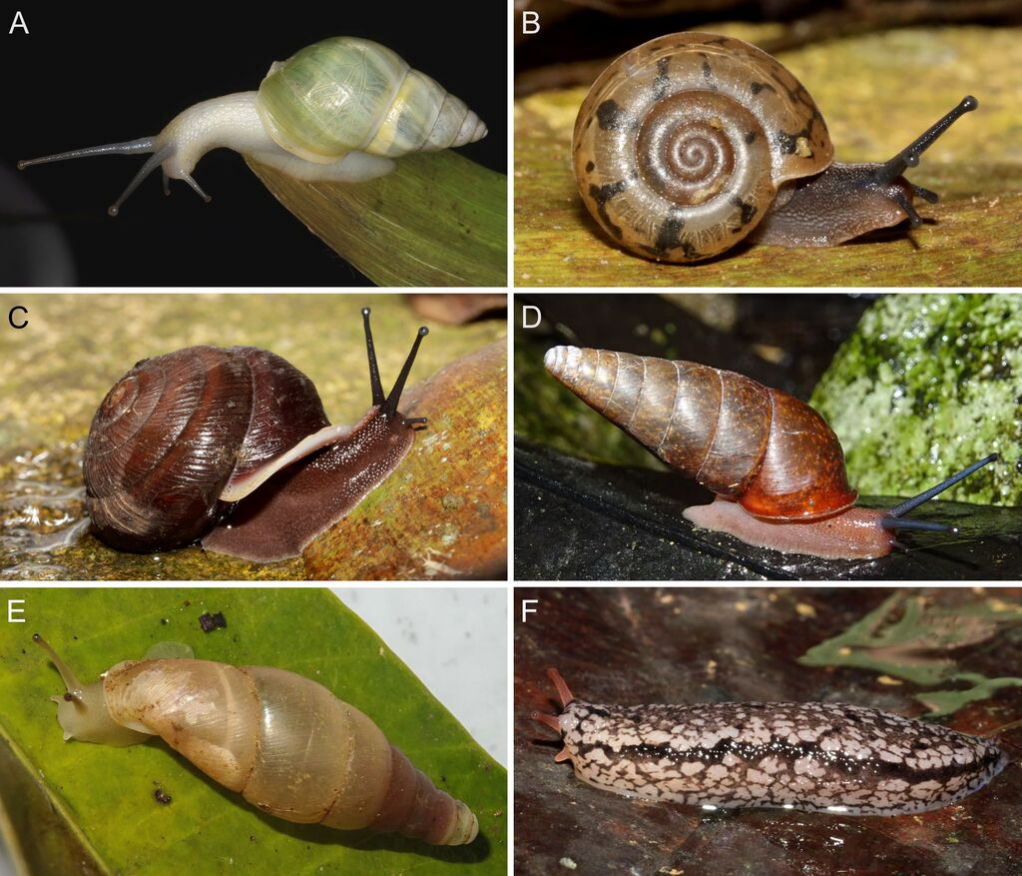
Photos of live gastropods from Cuc Phuong National Park. Specimens were collected in 2019 and are stored at the Museum für Naturkunde Berlin. **A** *Amphidromus roseolabiatus* Fulton, 1896; ZMB.Moll 269876. **B** *Chloritis* sp. A; ZMB.Moll 290322. **C** *Neocepolis merarcha* (Mabille, 1888); ZMB.Moll 290343a-1. **D** *Pseudobuliminus franzhuberi* Thach, 2018; ZMB.Moll 290349. **E** *Oospira miranda* (Loosjes & Loosjes-van Bemmel, 1973); ZMB.Moll 290401a-1. **F** *Meghimatium pictum* (Stoliczka, 1873); ZMB.Moll 291167-2.

**Figure 7.**
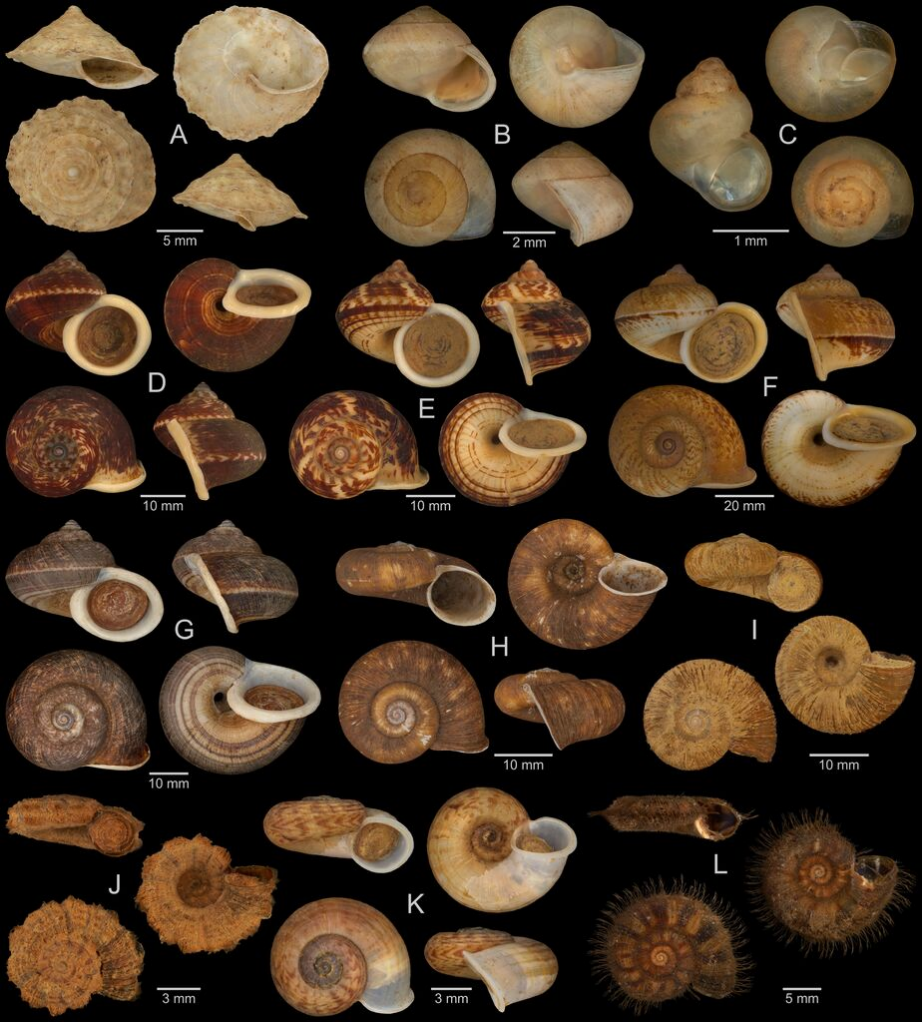
Terrestrial Mollusca specimens from Cuc Phuong National Park collected in 2019 and stored at the Museum für Naturkunde Berlin. **A** *Geotrochatella insignis* Dautzenberg, 1896; ZMB.Moll 291146d-1. **B** *Aphanoconia hungerfordiana tonkinensis* Möllendorff, 1909; ZMB.Moll 291143b-1. **C** *Georissa* sp. vi-4 *sensu* Vermeulen & Maassen 2003; ZMB.Moll 291161. **D** *Cyclophorus cucphuongensis* Oheimb, 2019; ZMB.Moll 291023b-1. **E** *Cyclophorus paracucphuongensis* Oheimb, 2019; ZMB.Moll 291035a-1. **F** *Cyclophorus* sp. (lineage 01 *sensu* Oheimb et al. 2018); ZMB.Moll 291037. **G** *Cyclophorus* sp. (lineage 08 *sensu* Oheimb et al. 2018); ZMB.Moll 291064b-1. **H** *Cyclotus danieli* (Morlet, 1886); ZMB.Moll 291073. **I** *Cyclotus danieli* (Morlet, 1886); ZMB.Moll 291078a. **J** *Scabrina* sp. A; ZMB.Moll 291097a-2. **K** *Scabrina* sp. B; ZMB.Moll 291105a-1. **L** *Scabrina* sp. vi-01 *sensu* Vermeulen & Maassen 2003; ZMB.Moll 291114.

**Figure 8.**
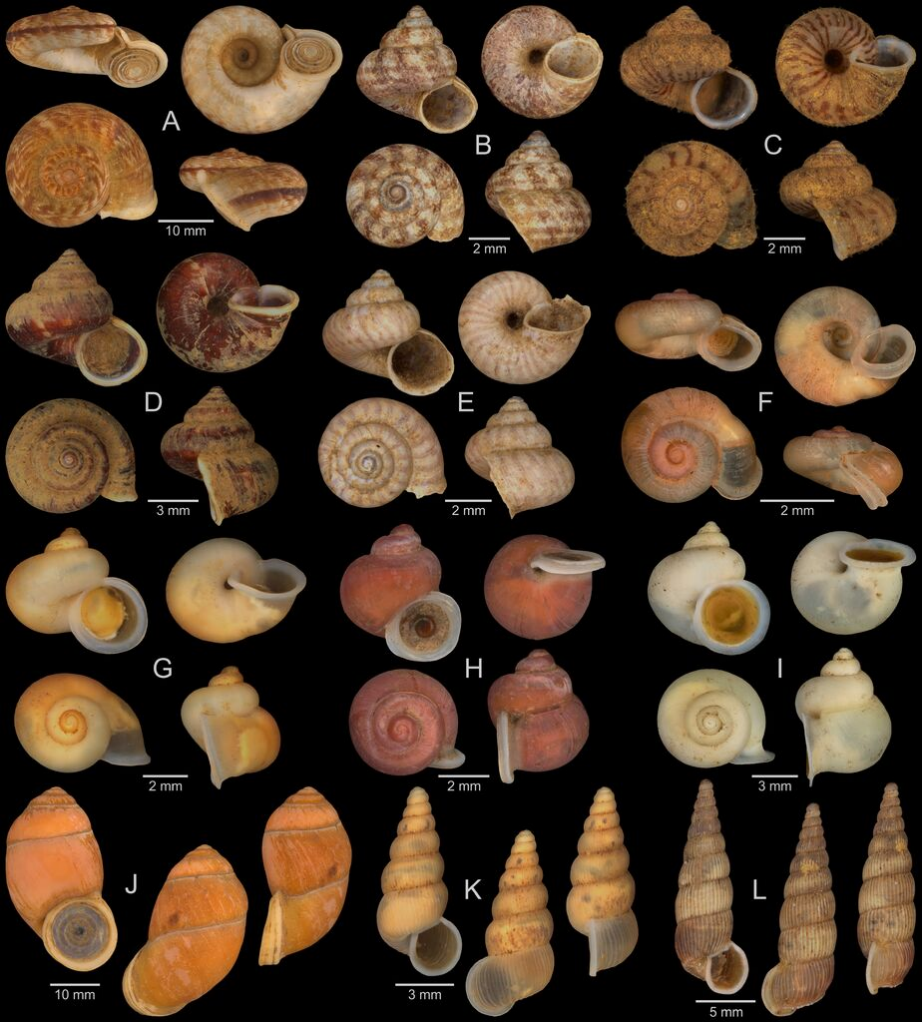
Terrestrial Mollusca specimens from Cuc Phuong National Park collected in 2019 and stored at the Museum für Naturkunde Berlin. **A** *Rhiostoma morleti* Dautzenberg & Fischer, 1906; ZMB.Moll 291092b-1. **B** *Lagocheilus hypselospirus* Möllendorff, 1901; ZMB.Moll 291082. **C** *Lagocheilus* sp. A; ZMB.Moll 291084a. **D** *Lagocheilus* sp. B; ZMB.Moll 291088. **E** *Lagocheilus* sp. C; ZMB.Moll 291090-1. **F** *Dicharax depressus* (Bavay & Dautzenberg, 1912); ZMB.Moll 290147c-1. **G** *Pincerna mouhoti* (Pfeiffer, 1863); ZMB.Moll 290169. **H** *Dioryx dongiensis* Varga, 1972; ZMB.Moll 290149b-1. **I** *Dioryx messageri* (Bavay & Dautzenberg, 1900); ZMB.Moll 290155-1. **J** *Pollicaria rochebruni* (Mabille, 1887); ZMB.Moll 291175. **K** *Pseudopomatias amoenus* Möllendorff, 1885; ZMB.Moll 291186. **L** *Vargapupa oharai* Páll-Gergely, 2015; ZMB.Moll 291209d.

**Figure 9.**
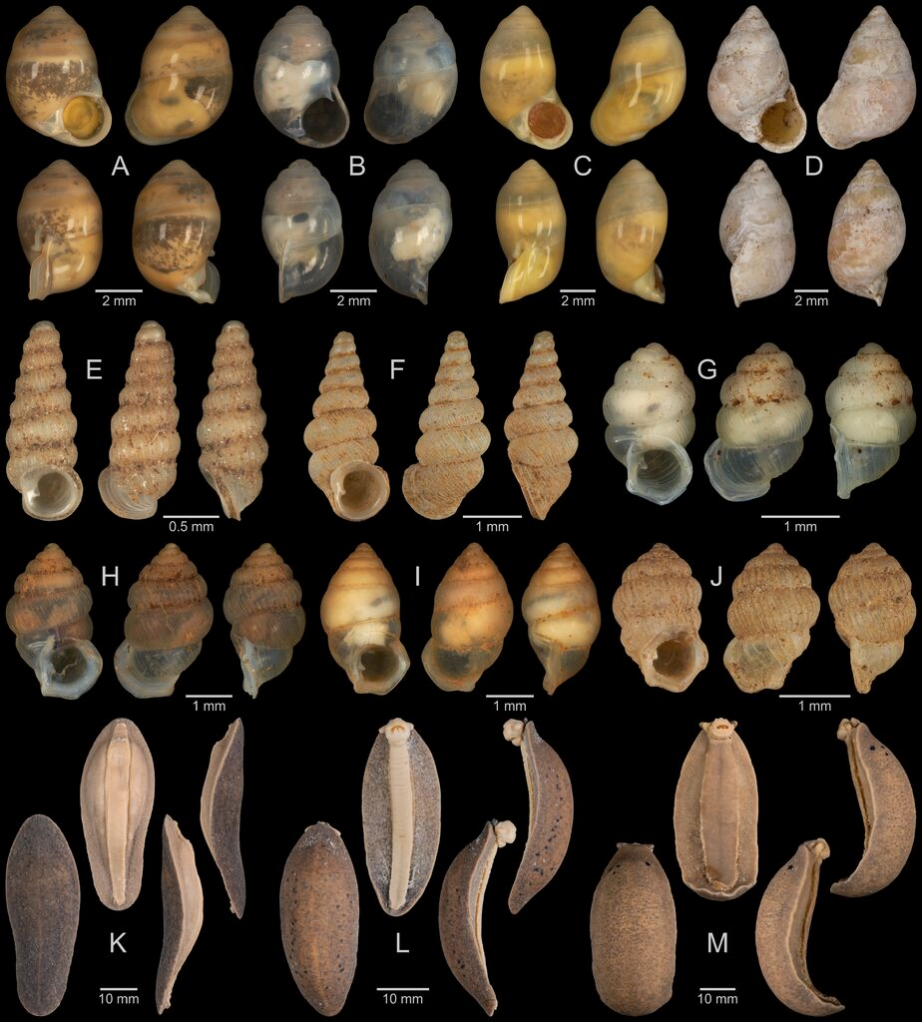
Terrestrial Mollusca specimens from Cuc Phuong National Park collected in 2019 and stored at the Museum für Naturkunde Berlin. **A** *Pupina anceyi* Bavay & Dautzenberg, 1899; ZMB.Moll 291188. **B** *Pupina dorri dorri* Dautzenberg, 1894; ZMB.Moll 291192. **C** *Pupina solidula* Möllendorff, 1901; ZMB.Moll 291198. **D** *Tylotoechus exclamationis* (Mabille, 1887); ZMB.Moll 291193. **E** *Diplommatina debilis* Bavay & Dautzenberg, 1904; ZMB.Moll 291117b-1. **F** *Diplommatina scolops* Möllendorff, 1901; ZMB.Moll 291118. **G** *Diplommatina* sp. vi-a2 *sensu* Vermeulen & Maassen 2003; ZMB.Moll 291122-1. **H** *Diplommatina* sp. vi-a3 *sensu* Vermeulen & Maassen 2003; ZMB.Moll 291127. **I** *Diplommatina* sp. vi-a4 *sensu* Vermeulen & Maassen 2003; ZMB.Moll 291129. **J** *Diplommatina tonkiniana* Jaeckel, 1950; ZMB.Moll 291130c-1. **K** Veronicellidae: gen. inc. sp. A; ZMB.Moll 265848. **L** Veronicellidae: gen. inc. sp. B; ZMB.Moll 266100. **M** Veronicellidae: gen. inc. sp. C; ZMB.Moll 265801.

**Figure 10.**
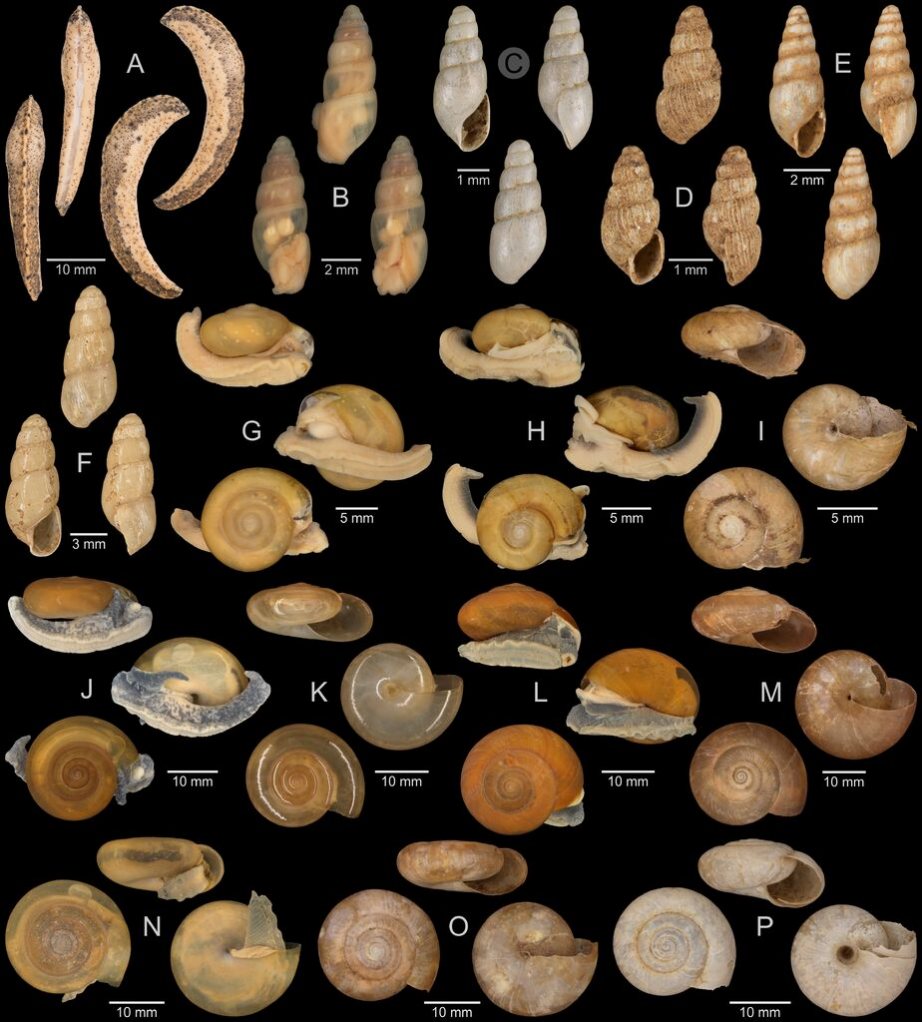
Terrestrial Mollusca specimens from Cuc Phuong National Park collected in 2019 and stored at the Museum für Naturkunde Berlin, and additional material collected in 1998 and stored in the personal collection of JJ Vermeulen (in grey, catalogue number prefix JJV). **A** *Atopos* sp. A; ZMB.Moll 266078. **B** *Allopeas clavulinum* (Potiez & Michaud, 1838); ZMB.Moll 290138. **C** *Allopeas gracile* (Hutton, 1834); JJV 7234 (part of lot). **D** *Curvella* sp. vi-1 *sensu* Vermeulen & Maassen 2003; ZMB.Moll 290142. **E** *Paropeas achatinaceum* (Pfeiffer, 1846); ZMB.Moll 290144b-1. **F** *Prosopeas ventrosulum* Bavay & Dautzenberg, 1909; ZMB.Moll 290146b-1. **G** *Macrochlamys* sp. vi-3 *sensu* Vermeulen & Maassen 2003; ZMB.Moll 290184. **H** *Macrochlamys* sp. A; ZMB.Moll 290187-1 (foot tissue cut off). **I** *Macrochlamys* sp. A; ZMB.Moll 290185. **J** *Macrochlamys despecta* (Mabille, 1887); ZMB.Moll 290189. **K** *Macrochlamys despecta* (Mabille, 1887); ZMB.Moll 290195b. **L** *Macrochlamys douvillei* Dautzenberg & Fischer, 1906; ZMB.Moll 290211a. **M** *Macrochlamys douvillei* Dautzenberg & Fischer, 1906; ZMB.Moll 290206c. **N** *Macrochlamys* sp. vi-2 *sensu* Vermeulen & Maassen 2003; ZMB.Moll 290218. **O** *Macrochlamys* sp. vi-2 *sensu* Vermeulen & Maassen 2003; ZMB.Moll 290215-1. **P** *Macrochlamys tenuigranosa* Dautzenberg, 1894; ZMB.Moll 290221.

**Figure 11.**
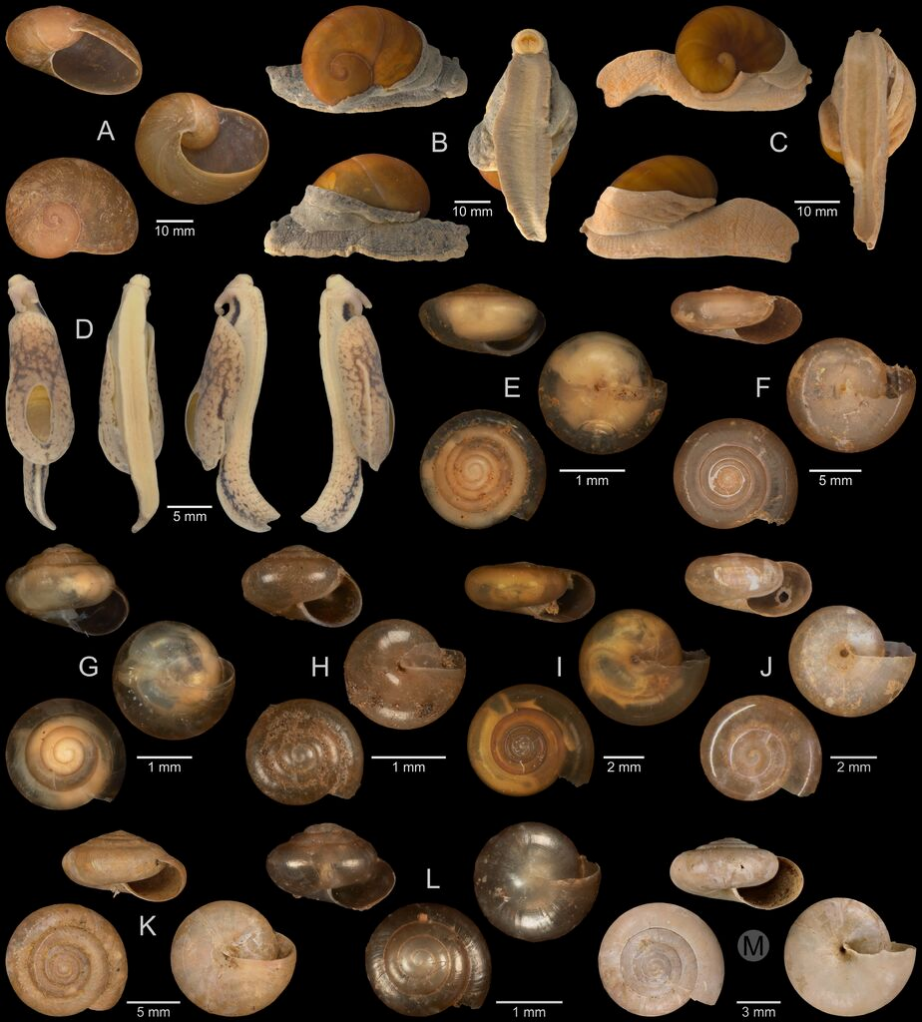
Terrestrial Mollusca specimens from Cuc Phuong National Park collected in 2019 and stored at the Museum für Naturkunde Berlin, and additional material collected in 1998 and stored in the personal collection of JJ Vermeulen (in grey, catalogue number prefix JJV). **A** *Megaustenia fragile* (Möllendorff, 1901); ZMB.Moll 290226b. **B** *Megaustenia fragile* (Möllendorff, 1901); ZMB.Moll 290222-3. **C** *Megaustenia messageri* (Bavay & Dautzenberg, 1909); ZMB.Moll 250152 (foot tissue cut off). **D** *Parmarion* sp. A; ZMB.Moll 290253. **E** *Microcystina* sp. A; ZMB.Moll 290232-1. **F** *Microcystina* sp. B; ZMB.Moll 290234. **G** *Microcystina* sp. C; ZMB.Moll 290235-1. **H** *Microcystina* sp. D; ZMB.Moll 290236b-1. **I** *Microcystina* sp. E; ZMB.Moll 290241a-1. **J** *Microcystina* sp. vi-b01 *sensu* Vermeulen & Maassen 2003; ZMB.Moll 290243. **K** *Microcystina* sp. vi-b03 *sensu* Vermeulen & Maassen 2003; ZMB.Moll 290246. **L** *Microcystina* sp. vi-b06 *sensu* Vermeulen & Maassen 2003; ZMB.Moll 290249. **M** *Microcystina* sp. vi-b10 *sensu* Vermeulen & Maassen 2003; JJV 7114.

**Figure 12.**
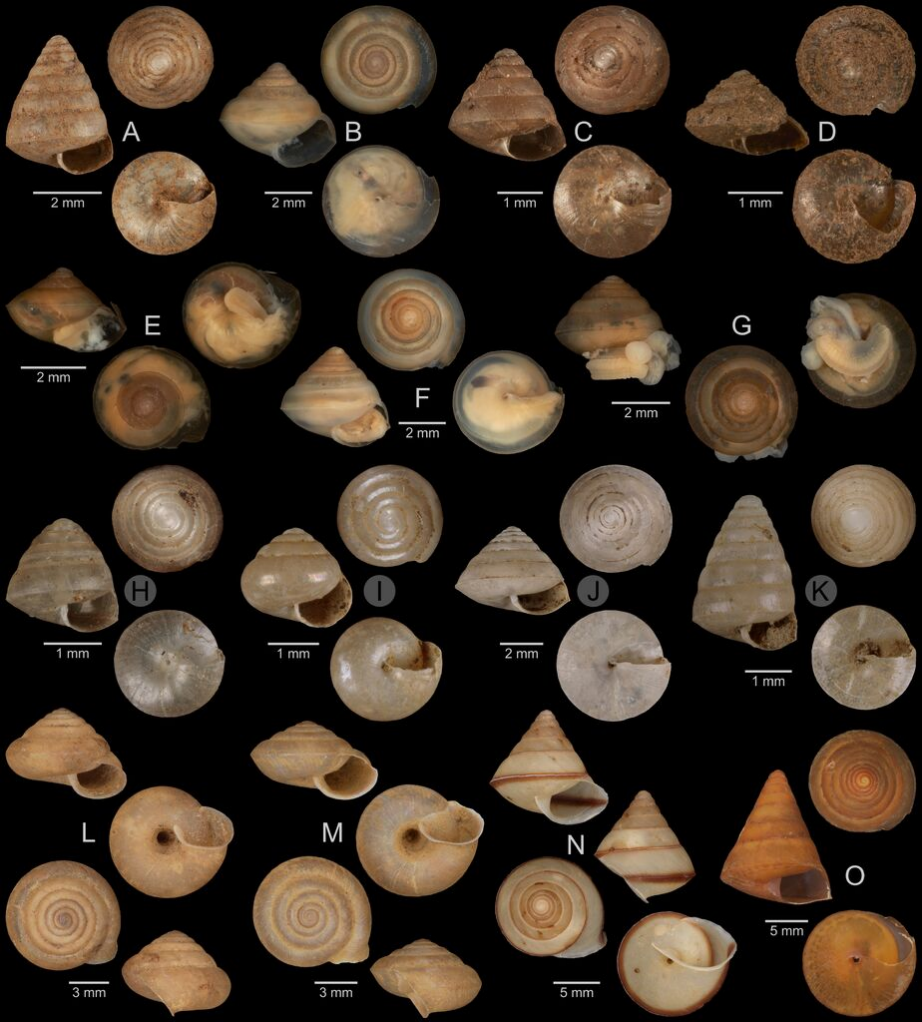
Terrestrial Mollusca specimens from Cuc Phuong National Park collected in 2019 and stored at the Museum für Naturkunde Berlin, and additional material collected in 1998 and stored in the personal collection of JJ Vermeulen (in grey, catalogue number prefix JJV). **A** *Kaliella haiphongensis* Dautzenberg, 1894; ZMB.Moll 290356b-1. **B** *Kaliella joubini* Dautzenberg & Fischer, 1905; ZMB.Moll 290359. **C** *Kaliella* sp. A; ZMB.Moll 290361b-1. **D** *Kaliella* sp. B; ZMB.Moll 290365-1. **E** *Kaliella* sp. C; ZMB.Moll 290368-1. **F** *Kaliella* sp. D; ZMB.Moll 290370. **G** *Kaliella* sp. E; ZMB.Moll 290371. **H** *Kaliella* sp. F; JJV 6976 (part of lot). **I** *Kaliella* sp. G; JJV 7027 (part of lot). **J** *Kaliella tongkingensis* Möllendorff, 1901; JJV 6988 (part of lot). **K** *Kaliella subelongata* Bavay & Dautzenberg, 1912; JJV 6963 (part of lot). **L** *Aegista coudeini* (Bavay & Dautzenberg, 1900); ZMB.Moll 290255d-1. **M** *Aegista pseudotrochula* (Bavay & Dautzenberg, 1909); ZMB.Moll 290267-1. **N** *Ganesella oxytropis* (Möllendorff, 1901); ZMB.Moll 290334b. **O** *Ganesella procera* Gude, 1902; ZMB.Moll 290339-1.

**Figure 13.**
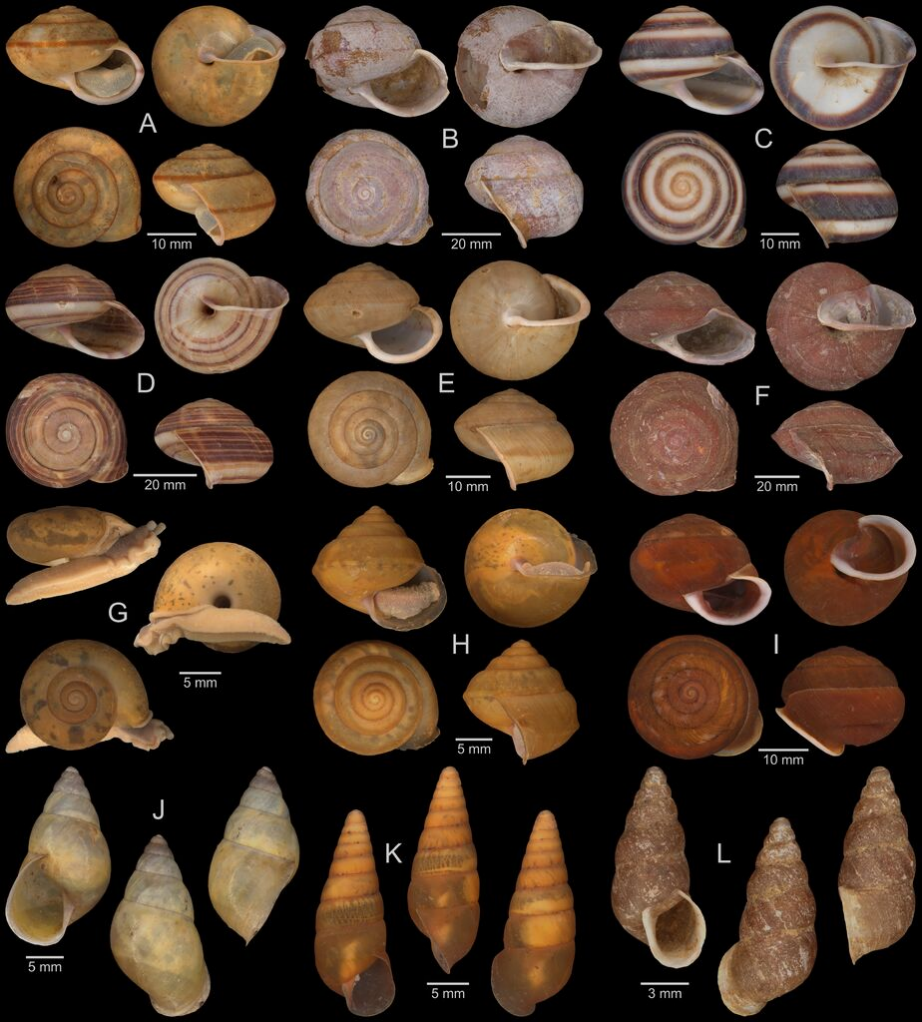
Terrestrial Mollusca specimens from Cuc Phuong National Park collected in 2019 and stored at the Museum für Naturkunde Berlin. **A** *Camaena billeti* (Fischer, 1898); ZMB.Moll 290272a-1. **B** *Camaena choboensis* (Mabille, 1889); ZMB.Moll 290279. **C** *Camaena chuongi* Thach, 2016; ZMB.Moll 290287b. **D** *Camaena onae* Thach, 2016; ZMB.Moll 290307-1. **E** *Camaena* sp. A; ZMB.Moll 290308. **F** *Camaena vanbuensis* Smith, 1896; ZMB.Moll 290318-1. **G** *Chloritis* sp. A; ZMB.Moll 290323a. **H** *Globotrochus onestera* (Mabille, 1887); ZMB.Moll 290341-1. **I** *Neocepolis merarcha* (Mabille, 1888); ZMB.Moll 290344. **J** *Amphidromus roseolabiatus* Fulton, 1896; ZMB.Moll 269883. **K** *Pseudobuliminus franzhuberi* Thach, 2018; ZMB.Moll 290349. **L** *Apoecus messageri* (Bavay & Dautzenberg, 1900); ZMB.Moll 291132b.

**Figure 14.**
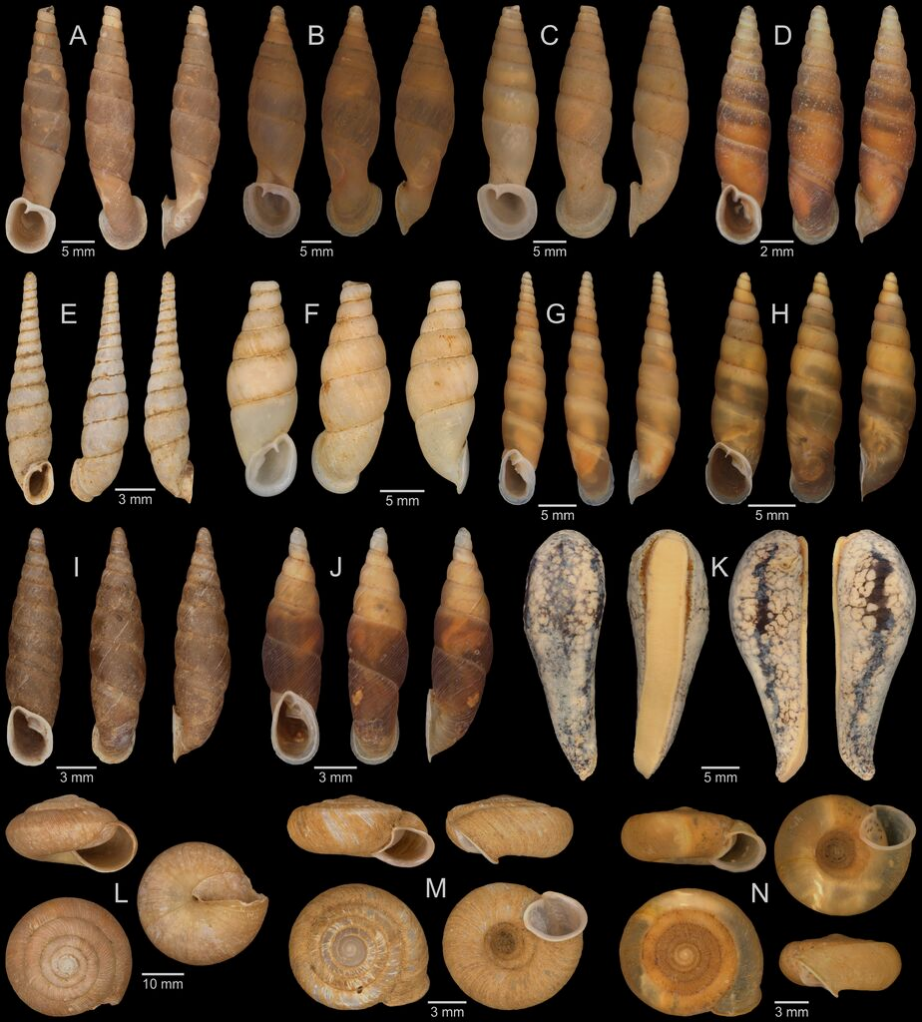
Terrestrial Mollusca specimens from Cuc Phuong National Park collected in 2019 and stored at the Museum für Naturkunde Berlin. **A** *Euryauchenia fischeri reticulata* (Nordsieck, 2002); ZMB.Moll 290377b. **B** *Megalauchenia proctostoma forceps* (Loosjes & Loosjes-van Bemmel, 1973); ZMB.Moll 290393b-1. **C** *Megalauchenia proctostoma proctostoma* (Mabille, 1889); ZMB.Moll 290395a-1. **D** *Oospira abstrusa abstrusa* (Szekeres, 1970); ZMB.Moll 290396a-1. **E** *Oospira eregia* (Szekeres, 1969); ZMB.Moll 290400-1. **F** *Oospira miranda* (Loosjes & Loosjes-van Bemmel, 1973); ZMB.Moll 290985e-1. **G** *Oospira vanbuensis* (Bavay & Dautzenberg, 1899); ZMB.Moll 290997a-1. **H** *Phaedusa dichroa* (Bavay & Dautzenberg, 1899); ZMB.Moll 291007-1. **I** *Phaedusa lypra* (Mabille, 1887); ZMB.Moll 291010c. **J** *Phaedusa stenothyra* Möllendorff, 1901; ZMB.Moll 291012b-2. **K** *Meghimatium pictum* (Stoliczka, 1873); ZMB.Moll 291165-1. **L** *Quantula* sp. 3 *sensu* Jirapatrasilp et al. 2021; ZMB.Moll 290183b. **M** *Gudeodiscus messageri raheemi* Páll-Gergely & Hunyadi, 2015; ZMB.Moll 291170c. **N** *Gudeodiscus phlyarius* (Mabille, 1887); ZMB.Moll 291173b.

**Figure 15.**
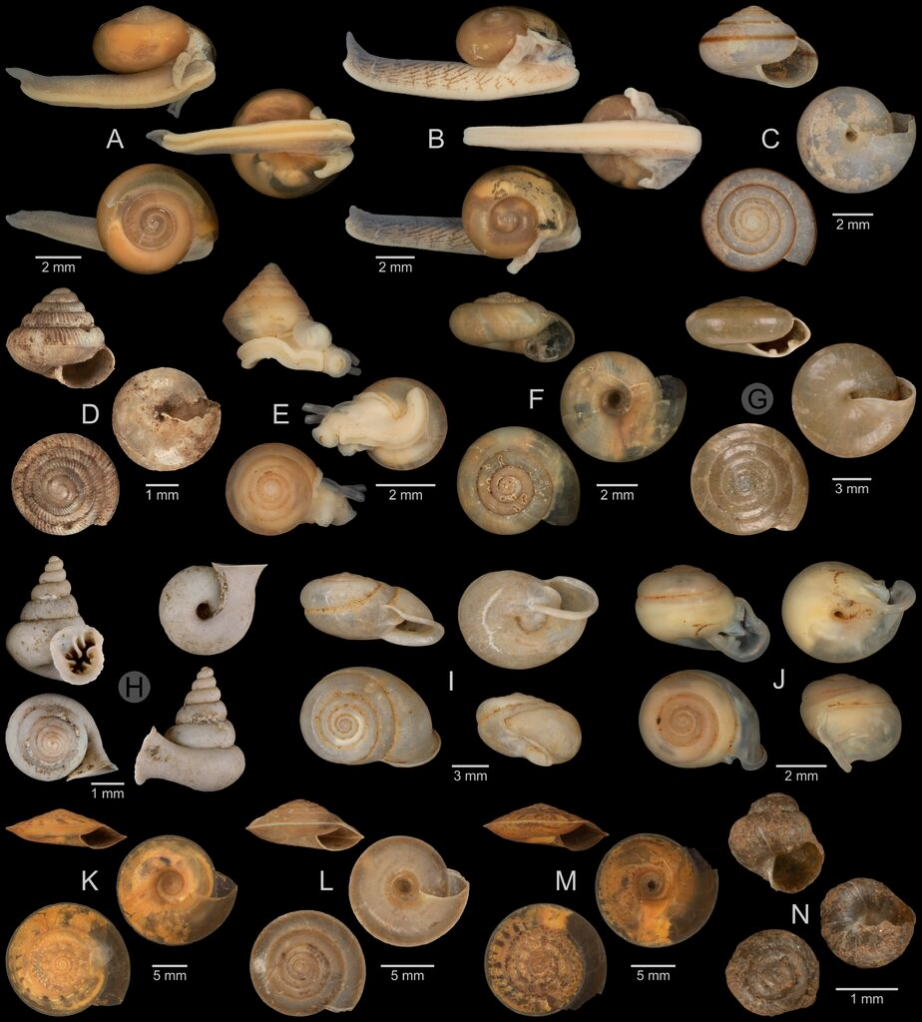
Terrestrial Mollusca specimens from Cuc Phuong National Park collected in 2019 and stored at the Museum für Naturkunde Berlin, and additional material collected in 1998 and stored in the personal collection of JJ Vermeulen (in grey, catalogue number prefix JJV). **A** *Durgella* sp. A; ZMB.Moll 291136. **B** *Durgella* sp. B; ZMB.Moll 291138. **C** *Chalepotaxis infantilis* (Gredler, 1881); ZMB.Moll 291135b. **D** *Rahula jucunda* (Bavay & Dautzenberg, 1912); ZMB.Moll 291139. **E** *Sitala confinis* (Möllendorff, 1901); ZMB.Moll 291141. **F** *Teraia contempta* (Bavay & Dautzenberg, 1909); ZMB.Moll 291142-1. **G** *Sesara* sp. vi-01 *sensu* Vermeulen & Maassen 2003; JJV 6909 (part of lot). **H** *Bensonella* sp. A; JJV 6273 (part of lot). **I** *Haploptychius diespiter* (Mabille, 1887); ZMB.Moll 291220-1. **J** *Perrottetia* sp. A; ZMB.Moll 291221. **K** *Trochomorpha paviei* (Morlet, 1885); ZMB.Moll 291232a. **L** *Trochomorpha saigonensis* (Crosse, 1867); ZMB.Moll 291235b-1. **M** *Trochomorpha* sp. A; ZMB.Moll 291239-1. **N** *Pupisoma dioscoricola* (Adams, 1845); ZMB.Moll 291241-1.

A total of 207 photos from 79 live individuals of 42 species were taken (data package; slightly edited example photos are shown in Figs 4, 5, 6). So far, material from 107 different (sub-)species has been digitised (a selection of slightly edited photos is shown in Figs 7, 8, 9, 10, 11, 12, 13, 14, 15; additional photos of eight taxa, for which no suitable 2019 material was available, show specimens from JJ Vermeulen’s set of the 1998 material stored in his personal collection). The compiled list of all terrestrial Mollusca known from Cuc Phuong National Park included 159 species and 1 additional subspecies (Table 4; see there also for additional literature). The mark-recapture analysis estimated a total species richness of 178 ± 7 shelled land mollusc species in Cuc Phuong National Park.

### Discussion

Our 2019 VIETBIO inventory work in Cuc Phuong National Park resulted in a substantial volume of new, collection-based data on the diversity and distribution of the local terrestrial mollusc fauna. Within the park, live gastropods and empty shells were often present in relatively high numbers at individual sampling sites. Such high abundances are typical for tropical limestone karst forests as they are rich in calcium (also indicated by the neutral to slightly alkaline soil pH values; Table 2, data package) and, thus, allow high population densities of shell-bearing gastropods (Schilthuizen et al. 2003). Based on our identification, we recorded a total of 116 species and 1 additional subspecies of terrestrial molluscs in the park. It should be noted, however, that many of the terrestrial gastropod species known from Vietnam are still poorly understood (Raheem et al. 2017), for instance, regarding their intraspecific variation in shell morphology and differences to other, similar species, while various taxa are yet undescribed and many others likely still await discovery. Amongst the species recorded by us in Cuc Phuong National Park were 47 only provisionally-named morphospecies. Our approach for assigning material to those was generally in keeping with common practices of species delimitation in molluscan taxonomy, and at least the shelled gastropods amongst them likely belong indeed to undescribed species. However, further data are required to verify our assumptions. Given that, in cases of doubt, we mostly applied established names instead of introducing provisional codes and regarded minor morphological differences as intraspecific variation, we probably have rather underestimated both, the number of yet undescribed species and the total number of species found. Furthermore, it should be noted that several genus-level groups present in Cuc Phuong National Park, in particular the genera *Microcystina* and *Kaliella* (Vermeulen et al. 2015, Pholyotha et al. 2023), are currently not well understood, while others, such as the genus *Macrochlamys*, require the study of further characters for unambiguous identification (Pholyotha et al. 2018). Thus, the generic assignment of the provisionally-named morphospecies was not always clear and should be re-assessed in future. Further studies on the recorded taxa, particularly such based on genetic data, could shed more light on their phylogenetic relationships, species boundaries and intraspecific variation. Such approaches could also help to identify yet undescribed morphologically cryptic diversity, which might have been well overlooked by our approach and could further increase the species number in the park. For instance, *Cyclophorus cucphuongensis* and *Cyclophorus paracucphuongensis* from Cuc Phuong National Park have been found to be indistinguishable if identification is solely based on shell characters and no distribution or DNA sequence data are considered (von Oheimb et al. 2019).

With more than 20 species of land snails and slugs that were newly recorded for the park during the 2019 VIETBIO inventory work, the list of terrestrial molluscs known from Cuc Phuong National Park now includes a total of 159 species and 1 additional subspecies (Table 4). In relation to other, comparably-sized forest regions in the global tropics, where the terrestrial mollusc fauna has been studied in some detail, the 222 km² Cuc Phuong National Park is placed amongst the most species-rich. For example, from the Kenyan Kakamega forest complex with an area of 265 km², Tattersfield (1996) reported 53 species of land snails and slugs. Boxnick et al. (2015) recorded 102 species for Nyungwe Forest National Park in Rwanda with an area of about 970 km². For the Panguana conservation area in Peru, which extends over 13 km², Wendebourg and Hausdorf (2019) stated a number of 75 known terrestrial gastropod species. With regard to studies from Southeast Asia, Liew et al. (2010), for example, collected 109 species of terrestrial molluscs in the 754 km² Kinabalu Park of Malaysian Borneo. A total of 55 terrestrial gastropod species were found from primary lowland rainforest, agroforest and teak plantations in the Kendeng Mountains of Java, Indonesia, by Nurinsiyah et al. (2016), while the most distant sampling sites were less than 12 km apart. Foon et al. (2017) collected 122 species of land snails on 12 limestone hills in Perak, Peninsular Malaysia, with the most distant sampling sites being ca. 80 km apart, while each hill contained between 39 and 63 species. For a non-limestone hill on Penang Island, Malaysia, Goh et al. (2025) listed a total of 66 known terrestrial gastropod species. In Vietnam, Vermeulen and Maassen (2003) recorded on the ca. 180 km² Cat Ba Island (Nguyen et al. 2010) and several islands off its northeast coast, an area that includes Cat Ba National Park, a total of 149 land snail species, which made it the area with the highest species number in their study. Do et al. (2020) recorded 56 terrestrial mollusc species from Ngoc Son-Ngo Luong Nature Reserve in Hoa Binh province, which covers an area of 193 km² and is located west of Cuc Phuong National Park on the same limestone range. From Pu Luong Nature Reserve in Thanh Hoa province, with an area of 177 km² and located further west on the same limestone range (Furey and Infield 2005), a total of 96 land snail species have been recorded by Vermeulen and Maassen (2003), which overlapped only to 35.2% with those listed by them for Cuc Phuong National Park. Given the considerably higher species diversity recorded for the nearby Cuc Phuong National Park, however, numbers for both nature reserves are probably underestimated (see also Vermeulen and Maassen 2003).

Insights in the ecological structuring of terrestrial gastropod communities might be gained from the proportion of non-eupulmonates to eupulmonates, which has been shown to differ considerably amongst biogeographical regions (see Schilthuizen 2011). From the terrestrial gastropod species known from Cuc Phuong National Park (Table 4), 47 species, representing about one-third, are non-eupulmonates (Cycloneritida, Architaenioglossa and Littorinimorpha), while the remaining 112 belong to the eupulmonates (Ellobiida, Systellommatophora and Stylommatophora). This is in general concordance with other tropical regions in Southeast and South Asia, which contain much higher proportions of non-eupulmonates compared to tropical regions in Africa, where the terrestrial malacofauna can be almost exclusively represented by eupulmonates (summarised in Schilthuizen 2011). These patterns may result from differences amongst the groups’ evolutionary history and ecology, with non-eupulmonates being generally less tolerant to drought, temperature extremes and disturbance (Solem 1974, Schilthuizen et al. 2005, Schilthuizen 2011, Schilthuizen et al. 2020). In Cuc Phuong National Park, only five species of slugs have been recorded, which account for ca. 3% of the park’s known terrestrial gastropod fauna, while most of these species were represented only by few individuals in the 2019 collection (Fig. 3). This pattern appears to be similar to other regions of tropical forest in Asia, where slugs form a relatively minor component of the terrestrial malacofauna (e.g. Schilthuizen and Liew 2008, Inkhavilay et al. 2019, Thilakarathne et al. 2024).

A total of 43 terrestrial mollusc species known from Cuc Phuong National Park were not recorded during our 2019 inventory work (see Table 4). It must, therefore, be assumed that additional sampling would have resulted in the recording of a considerable number of further species. Even though sampling effort and strategy differed amongst sites, this is also evident from the significant proportion of taxa that were only found at few localities, with ca. 26% of all (sub-)species being recorded only from one site each (Fig. 3). Furthermore, the abundance of the different taxa in the collection varied considerably, with many species being represented in very low individual numbers, ca. 15% only by a single individual (Fig. 3). Many species known from the park are, thus, probably rare or unevenly distributed throughout the area and further ones might have been missed by the surveys carried out so far. Mark-recapture approaches can be utilised for estimating an area’s total species richness, including such species that could not actually be recorded (Boulinier et al. 1998). The analysis conducted here was based on the shelled land mollusc species found in the park during the 1998 and 2019 surveys. The heterogeneity model *M*_h_ with the associated jackknife estimator assumes that species-specific detection probabilities remained constant between the independent capture events (Burnham and Overton 1979). In calcium-rich regions, such as Cuc Phuong National Park, the durable gastropod shells allow the recording of species regardless of possible population fluctuations (see Schilthuizen 2011), and with the approach used in each of the two surveys, virtually all shelled mollusc species would generally be detectable. While the 1998 sampling, however, had less sampling sites and a stronger focus on soil sampling (see Vermeulen and Maassen 2003), the latter likely increasing the probability of finding small species, the applied model has been considered as generally robust to deviations from model assumptions (see Boulinier et al. 1998) and appropriate for species incidence data from multi-observer surveys with heterogeneous sampling conditions (Jiguet et al. 2005). The analysis predicted that a total of 178 ± 7 shelled land mollusc species occur in Cuc Phuong National Park. When adding the six species of (semi-)slugs, which we found in 2019, but disregarded for the analysis, at least 184 ± 7 terrestrial gastropod species can be expected, which would exceed the total number of 159 species known from the park by ca. 11-20%. While these predicted numbers should be considered rough estimates, such statistical approaches can be valuable tools for assessing the actual terrestrial mollusc species richness in tropical regions, where comprehensive inventories are challenging to conduct.

While knowledge on the exact distribution of taxa within Cuc Phuong National Park remains limited, the available data indicate that the park’s high overall number of terrestrial mollusc species results from a combined effect of the three patterns that can account for a high species diversity of land snails in an area according to Solem (1984): allopatric diversity, mosaic diversity and sympatric diversity. Allopatric diversity of species with small distribution ranges has been found for representatives of the genus *Cyclophorus*, which replace each other in different parts of Cuc Phuong National Park (von Oheimb et al. 2019). During the 2019 inventory work, we were able to record various further taxa only from certain parts of the park (see data package), while others showed a wider distribution. Such patterns of restricted distribution emphasise the importance of a geographically small-scale sampling during inventories of terrestrial molluscs in tropical forests, even across relatively homogenous study areas of limited size. In Cuc Phuong National Park, future studies should pay particular attention to the peripheral areas of the park. Both, mosaic diversity, where species are restricted to particular habitats within a heterogeneous system, and sympatric diversity, where species inhabit exactly the same place, could generally apply to cases where species were found to co-occur in the same area within Cuc Phuong National Park. While these two patterns are not always easy to distinguish and also depend on the scale (see Seddon et al. 2005), evidence for both may be derived from our microhabitat data. It should be acknowledged, however, that we distinguished only four relatively broad microhabitat categories (which were also not always unambiguous, for example, in cases of leaf litter in rock crevices or soil samples that could contain small ground-dwelling specimens) and that our sampling strategy was not quantitative. Species that showed overlapping distribution areas within the park, while being typically found in different microhabitats, provide evidence for mosaic diversity. For example, the majority of live *Dioryx* spp. specimens were found on vegetation, most *Geotrochatella insignis* individuals occurred on rock and most *Pollicaria rochebruni* individuals on the ground. The co-occurrence of species in the same microhabitat might represent sympatric diversity; however, our data are not sufficient to discriminate between patterns of actual syntopy and small-scale habitat differentiation. While the exact habitat requirements and niche differentiation of the park’s taxa need further study, it should, moreover, be kept in mind that habitat preferences might change over time, being influenced, for example, by daytime and season (see Cook 2001).

The present paper and its corresponding data package underline that as a protected area, Cuc Phuong National Park preserves a multitude of terrestrial mollusc species. Many of the taxa that inhabit the park are probably endemic to the region (see Vermeulen and Maassen 2003). While we do not aim to provide an identification guide, the photos presented with this publication might nevertheless facilitate the determination of the park’s gastropod species. The 2019 collection, which is stored at the Museum für Naturkunde Berlin and at the Institute of Ecology and Biological Resources, Ha Noi, allows the re-examination of our results. While some of the material has already been included in recent studies (Sulikowska-Drozd et al. 2022, Do et al. 2024), the collection, together with the data published here, can provide the basis for further research on the biodiversity, systematics and evolution of the region’s terrestrial mollusc fauna.

## Supplementary Material

39F4EB76-9B39-5635-A50A-2A69DEC0C2FF 10.3897/BDJ.13.e163277.suppl1 Supplementary material 1 Terrestrial Mollusca of Cuc Phuong National Park, Vietnam – Results from the 2019 VIETBIO inventory work. Occurrence data Data type CSV file Brief description Relevant occurrence data from the Darwin Core Archive of the data package (corresponds to version 1.0). Other than in the Darwin Core Archive, each specimen is represented only once and few additional data on life stage and identification uncertainty for remaining material from lots where specimens were separated are included. File: oo_1385983.csv https://binary.pensoft.net/file/1385983 Parm Viktor von Oheimb, Anna Sulikowska-Drozd, Thuy Dieu Dinh, Nora Lentge-Maaß, Tu Van Do, Katharina C. M. von Oheimb

## Figures and Tables

**Table 1.** List of nominal species-group taxa of terrestrial Mollusca with a type locality in Cuc Phuong National Park. Note that, contrary to their original descriptions (Nordsieck 2010), the type localities of *Ptychauchenia panhai* Nordsieck, 2010 and *Neniauchenia tonkinensis* Nordsieck, 2010 (*Grandinenia tonkinensis* (Nordsieck, 2010) following Páll-Gergely and Szekeres 2017) are not located in Cuc Phuong National Park (corrected in Nordsieck 2016).

**Family**	**Nominal species-group taxon**	**Original description reference**
Cyclophoridae	Chamalycaeus (Dicharax) fractus Varga, 1974; herein regarded as synonym of *Metalycaeus heudei* (Bavay & Dautzenberg, 1900)^1^	Varga (1974)
Cyclophoridae	*Cyclophorus cucphuongensis* Oheimb, 2019	von Oheimb et al. (2019)
Cyclophoridae	*Cyclophorus paracucphuongensis* Oheimb, 2019	von Oheimb et al. (2019)
Cyclophoridae	*Cyclophorus takumisaitoi* Hirano, 2019	von Oheimb et al. (2019)
Cyclophoridae	*Dioryx dongiensis* Varga, 1972	Varga (1972)
Cyclophoridae	*Dioryx pocsi* Varga, 1972	Varga (1972)
Pupinidae	*Vargapupa oharai* Páll-Gergely, 2015	Páll-Gergely et al. (2015a)
Clausiliidae	*Euphaedusa abstrusa* Szekeres, 1970; herein *Oospira abstrusa* (Szekeres, 1970)^2^	Szekeres (1970)
Clausiliidae	*Phaedusa umbratica* Szekeres, 1970; herein regarded as synonym of *Oospira vanbuensis* (Bavay & Dautzenberg, 1899)^3^	Szekeres (1970)
Hypselostomatidae	*Angustopila oostoma* Páll-Gergely & Vermeulen, 2023	Páll-Gergely et al. (2023)
Hypselostomatidae	*Angustopila vandevenderi* Páll-Gergely & Jochum, 2023	Páll-Gergely et al. (2023)

^1^Taxonomy follows Páll-Gergely et al. (2017b). ^2^Taxonomy follows Grego et al. (2014). ^3^Taxonomy follows Nordsieck (2011).

**Table 2.** List of sampling events (at sampling points, or along sampling transects with a starting and end point) from the 2019 VIETBIO inventory work of terrestrial Mollusca in Cuc Phuong National Park, including eventID, event date and time, sampling protocol(s) used, locality information, collector(s) (authors indicated by their initials) and soil pH. Coordinates are based on the WGS 84 (EPSG:4326) standard.

**eventID**	**Event date and time**	**Sampling protocol**	**Latitude, longitude (GPS accuracy)**	**Elevation above sea level**	**Locality in Cuc Phuong National Park**	**Collector(s)**	**Soil pH**	**Remarks**
VN2019-001	29 Apr 2019, 20:15	hand collecting	20.2451, 105.7160 (9 m)	152 m	Carnivore and Pangolin Education Centre	PVvO, KCMvO, AS-D		
VN2019-002	29 Apr 2019, 22:00	hand collecting	20.2502, 105.7146 (11 m)	155 m	Headquarter	PVvO, KCMvO, Sarah Ehlers, Christoph L. Häuser		
VN2019-003	30 Apr 2019, 09:50-13:00	hand collecting, soil sieving	20.3525, 105.5192 (12 m)	101 m	Forest west of Buoi river	PVvO, KCMvO, AS-D, TDD, TVD	7.42	
VN2019-004	01 May 2019	hand collecting	20.3524, 105.5199	81 m	Forest near cave west of Buoi river	TVD		
VN2019-005	30 Apr 2019, 13:00-14:22	hand collecting, soil sieving	start: 20.3525, 105.5192 (12 m); end: 20.3523, 105.5202 (17 m)	start: 101 m; end: 71 m	Through forest west of Buoi river	PVvO, KCMvO, AS-D, TDD, TVD	7.50	soil sample from end point of transect
VN2019-006	01 May 2019, 09:52-10:49	hand collecting	20.3833, 105.5226 (16 m)	89 m	Edge of forest near Khanh village east of Buoi river	PVvO, KCMvO, AS-D, TDD		
VN2019-008	01 May 2019, 11:15-13:20	hand collecting, soil sieving	start: 20.3817, 105.5200 (6 m); end: 20.3819, 105.5193 (6 m)	start: 102 m; end: 132 m	Through forest near Khanh village east of Buoi river	PVvO, KCMvO, AS-D, TDD	7.68	soil sample from starting point of transect
VN2019-009	03 May 2019, 10:06-13:21	hand collecting	20.2948, 105.6689 (6 m)	311 m	Forest near Cave of Prehistoric Man	PVvO, KCMvO		
VN2019-010	03 May 2019, 10:30-13:11	hand collecting	20.2949, 105.6702 (10 m)	359 m	Forest near Cave of Prehistoric Man	AS-D, TDD		
VN2019-011	03 May 2019, 13:25-14:07	hand collecting	start: 20.2948, 105.6689 (6 m); end: 20.2931, 105.6670 (10 m)	start: 311 m; end: 262 m	Through forest near Cave of Prehistoric Man	PVvO, KCMvO, AS-D, TDD		
VN2019-012	03 May 2019, 15:15	hand collecting	20.2907, 105.6685 (5 m)	250 m	Road near Cave of Prehistoric Man	AS-D		
VN2019-013	04 May 2019, 07:45 to 10 May 2019, 13:00	hand collecting	20.2499, 105.7144 (3 m)	153 m	Headquarter	PVvO, KCMvO, AS-D		discontinuous sampling period
VN2019-014	04 May 2019, 09:48-11:23	hand collecting, soil sieving	20.2507, 105.7127 (4 m)	208 m	Forest at observation hide near Headquarter	PVvO, KCMvO, AS-D	6.78	
VN2019-015	04 May 2019, 11:25-12:17	hand collecting	start: 20.2507, 105.7127 (4 m); end: 20.2507, 105.7141 (3 m)	start: 208 m; end: 157 m	Along forest path to observation hide near Headquarter	PVvO, KCMvO, AS-D		
VN2019-017	05 May 2019, 09:15-10:15	hand collecting	20.3143, 105.6421 (9 m)	287 m	Forest path to Ancient Tree	AS-D, TDD		
VN2019-018	05 May 2019, 10:45-12:09	hand collecting	20.3168, 105.6401 (11 m)	289 m	Forest near Ancient Tree	AS-D, TDD		
VN2019-019	05 May 2019, 12:10-13:01	hand collecting	start: 20.3168, 105.6401 (11 m); end: 20.3163, 105.6350 (13 m)	start: 289 m; end: 287 m	Through forest near Ancient Tree	AS-D, TDD		
VN2019-020	02 May 2019, 21:30 to 04 May 2019, 22:19	hand collecting	20.25380, 105.71039	164 m	Bridge over ditch between Headquarter and Mac Lake	AS-D		discontinuous sampling period
VN2019-021	03 May 2019	hand collecting	20.2911, 105.6686 (27 m)	251 m	Road near Cave of Prehistoric Man	Christoph L. Häuser		
VN2019-022	06 May 2019 to 09 May 2019	hand collecting	start: 20.3482, 105.5977 (9 m); end: 20.3543, 105.5854 (3 m)	start: 379 m; end: 378 m	Along Park Centre	PVvO, KCMvO, AS-D, TDD, VIETBIO team		discontinuous sampling period
VN2019-023	06 May 2019, 19:30	hand collecting	20.34977, 105.59646	398 m	Forest near Park Centre	AS-D		
VN2019-024	07 May 2019, 09:59-11:36	hand collecting, soil sieving	20.3582, 105.5812 (6 m)	390 m	Forest near small stream west of Park Centre	PVvO, KCMvO, AS-D, TDD	6.86	
VN2019-025	07 May 2019, 12:52-14:39	hand collecting, soil sieving	20.3570, 105.5819 (9 m)	383 m	Forest west of Park Centre	PVvO, KCMvO, AS-D, TDD	6.97	
VN2019-026	08 May 2019, 08:51-13:10	hand collecting	start: 20.3355, 105.6100 (4 m); end: 20.3327, 105.6069 (14 m)	start: 309 m; end: 410 m	Through forest east of Park Centre	PVvO, KCMvO, AS-D, TDD		
VN2019-027	08 May 2019, 10:06-12:19	hand collecting	20.3331, 105.6081 (6 m)	386 m	Forest east of Park Centre	PVvO, KCMvO		
VN2019-028	08 May 2019, 10:24-12:10	hand collecting	20.3327, 105.6069 (14 m)	410 m	Forest east of Park Centre	AS-D, TDD		
VN2019-029	08 May 2019, 13:29-14:00	hand collecting	start: 20.3388, 105.6077 (10 m); end: 20.3464, 105.6005 (7 m)	start: 337 m; end: 379 m	Along road east of Park Centre	PVvO, KCMvO		
VN2019-030	08 May 2019, 15:00-16:30	hand collecting	start: 20.3504, 105.6032 (13 m); end: 20.3584, 105.6017 (20 m)	start: 454 m; end: 516 m	Along forest path to Palace Cave	AS-D		
VN2019-031	06 May 2019	hand netting	20.34746, 105.59924	365 m	Road east of Park Centre	Sarah Ehlers		
VN2019-032	09 May 2019, 10:20-12:53	hand collecting	start: 20.3526, 105.5922 (7 m); end: 20.3530, 105.5922 (14 m)	start: 416 m; end: 431 m	Through forest near Park Centre	PVvO, KCMvO		
VN2019-033	07 May 2019	hand collecting	20.3580, 105.5809 (32 m)	399 m	Forest near small stream west of Park Centre	Thomas von Rintelen		
VN2019-034	09 May 2019	hand collecting	20.28250, 105.67628	232 m	Road east of Cave of Prehistoric Man	TVD		
VN2019-035	09 May 2019, 09:30-10:45	hand collecting	20.2916, 105.6661 (17 m)	280 m	Forest near Cave of Prehistoric Man	AS-D, TDD		
VN2019-036	09 May 2019, 11:30-12:30	hand collecting, soil sieving	20.2916, 105.6667 (18 m)	284 m	Forest near Cave of Prehistoric Man	AS-D, TDD	7.04	

**Table 3.** List of terrestrial molluscs recorded during the 2019 VIETBIO inventory work in Cuc Phuong National Park, including family and (sub-)species name, literature used for taxonomic identification, respective recording sites, and information on figures in this paper and the data package.

**Family**	**(Sub-)Species**	**Literature for identification**	**Recording sites**	**Figures**
**Order Cycloneritida**				
Helicinidae	*Aphanoconia hungerfordiana tonkinensis* Möllendorff, 1909	Wagner (1911)	VN2019-008	Fig. 7B, data package
Helicinidae	*Geotrochatella insignis* Dautzenberg, 1896	Dautzenberg (1896), Wagner (1911), Vermeulen and Maassen (2003)^1^	VN2019-003, VN2019-005, VN2019-008, VN2019-009, VN2019-010, VN2019-015, VN2019-017, VN2019-018, VN2019-024, VN2019-026, VN2019-027, VN2019-028, VN2019-032, VN2019-035, VN2019-036	Fig. 7A, data package (see also Fig. 4A)
Hydrocenidae	*Georissa* sp. vi-4 *sensu* Vermeulen & Maassen 2003	Vermeulen and Maassen (2003) ^1^	VN2019-003, VN2019-005, VN2019-024, VN2019-025, VN2019-032	Fig. 7C
**Order Architaenioglossa**				
Cyclophoridae	*Cyclophorus cucphuongensis* Oheimb, 2019	Vermeulen and Maassen (2003)^1^, von Oheimb et al. (2019)	VN2019-009, VN2019-010, VN2019-011, VN2019-017, VN2019-018, VN2019-020, VN2019-022, VN2019-025, VN2019-026, VN2019-027, VN2019-028, VN2019-029, VN2019-030, VN2019-032, VN2019-034, VN2019-035, VN2019-036	Fig. 7D
Cyclophoridae	*Cyclophorus paracucphuongensis* Oheimb, 2019	von Oheimb et al. (2019)	VN2019-003, VN2019-004, VN2019-005	Fig. 7E, data package (see also Fig. 4B)
Cyclophoridae	*Cyclophorus* sp. (lineage 01 *sensu* Oheimb et al. 2018)	Vermeulen and Maassen (2003)^1^, von Oheimb et al. (2018)	VN2019-003, VN2019-005, VN2019-006, VN2019-008, VN2019-009^2,3^, VN2019-010, VN2019-014, VN2019-017, VN2019-018^2^, VN2019-022^2^, VN2019-025, VN2019-026, VN2019-027, VN2019-028, VN2019-035	Fig. 7F, data package
Cyclophoridae	*Cyclophorus* sp. (lineage 08 *sensu* Oheimb et al. 2018)	Vermeulen and Maassen (2003)^1^, von Oheimb et al. (2018)	VN2019-003, VN2019-005, VN2019-008, VN2019-009, VN2019-010, VN2019-014^2^, VN2019-015, VN2019-017, VN2019-018, VN2019-022, VN2019-024, VN2019-025, VN2019-026, VN2019-027, VN2019-028, VN2019-029, VN2019-030, VN2019-035, VN2019-036^2^	Fig. 7G, data package
Cyclophoridae	*Cyclotus danieli* (Morlet, 1886)	Morlet (1886b), Kobelt and von Möllendorff (1897), Vermeulen and Maassen (2003)^1^	VN2019-003^2,3^, VN2019-005^2^, VN2019-009, VN2019-010, VN2019-026, VN2019-027, VN2019-028, VN2019-035, VN2019-036	Fig. 7H, Fig. 7I
Cyclophoridae	*Dicharax depressus* (Bavay & Dautzenberg, 1912)	Bavay and Dautzenberg (1912), Vermeulen and Maassen (2003)^1^, Páll-Gergely et al. (2017b), Páll-Gergely et al. (2020c)	VN2019-014, VN2019-015	Fig. 8F
Cyclophoridae	*Dioryx dongiensis* Varga, 1972	Varga (1972), Vermeulen and Maassen (2003)^1^, Páll-Gergely et al. (2020c)	VN2019-009, VN2019-019, VN2019-025, VN2019-026, VN2019-027, VN2019-028	Fig. 8H, data package
Cyclophoridae	*Dioryx messageri* (Bavay & Dautzenberg, 1900)	Bavay and Dautzenberg (1900a), Bavay and Dautzenberg (1900b), Vermeulen and Maassen (2003)^1^, Páll-Gergely et al. (2020c)	VN2019-003, VN2019-005, VN2019-011, VN2019-017, VN2019-018, VN2019-019, VN2019-024, VN2019-025, VN2019-026, VN2019-027, VN2019-028, VN2019-029, VN2019-032, VN2019-035	Fig. 8I, data package (see also Fig. 4C)
Cyclophoridae	*Lagocheilus hypselospirus* Möllendorff, 1901	von Möllendorff (1901a), Zilch (1955), Vermeulen and Maassen (2003)^1^	VN2019-003, VN2019-005, VN2019-008, VN2019-025	Fig. 8B
Cyclophoridae	*Lagocheilus* sp. A	Vermeulen and Maassen (2003)^1^, Hirano et al. (2019) [generic assignment]	VN2019-003^2,3^, VN2019-008, VN2019-009, VN2019-014^2,3^, VN2019-024	Fig. 8C
Cyclophoridae	*Lagocheilus* sp. B	Vermeulen and Maassen (2003)^1^, Hirano et al. (2019) [generic assignment]	VN2019-009, VN2019-026	Fig. 8D
Cyclophoridae	*Lagocheilus* sp. C	Vermeulen and Maassen (2003)^1^, Hirano et al. (2019) [generic assignment]	VN2019-003	Fig. 8E
Cyclophoridae	*Pincerna mouhoti* (Pfeiffer, 1863)	Pfeiffer (1863), Vermeulen and Maassen (2003)^1^, Páll-Gergely et al. (2020c), Páll-Gergely (2023)	VN2019-010, VN2019-025, VN2019-026, VN2019-027	Fig. 8G, data package
Cyclophoridae	*Rhiostoma morleti* Dautzenberg & Fischer, 1906	Dautzenberg and Fischer (1906), Tongkerd et al. (2023)	VN2019-005, VN2019-017, VN2019-035, VN2019-036	Fig. 8A, data package^4^
Cyclophoridae	*Scabrina* sp. A	Vermeulen and Maassen (2003)^1^, Foon and Marzuki (2022) [generic assignment]	VN2019-003, VN2019-005, VN2019-008, VN2019-014, VN2019-015, VN2019-017, VN2019-022, VN2019-024, VN2019-025, VN2019-036^3^	Fig. 7J
Cyclophoridae	*Scabrina* sp. B	Vermeulen and Maassen (2003)^1^, Foon and Marzuki (2022) [generic assignment]	VN2019-003, VN2019-005, VN2019-008, VN2019-026, VN2019-028, VN2019-035, VN2019-036	Fig. 7K, data package
Cyclophoridae	*Scabrina* sp. vi-01 *sensu* Vermeulen & Maassen 2003	Vermeulen and Maassen (2003) ^1^	VN2019-009, VN2019-011, VN2019-030	Fig. 7L, data package (see also Fig. 4D)
Diplommatinidae	*Diplommatina debilis* Bavay & Dautzenberg, 1904	Bavay and Dautzenberg (1904), Vermeulen and Maassen (2003)^1^	VN2019-003, VN2019-008	Fig. 9E
Diplommatinidae	*Diplommatina scolops* Möllendorff, 1901	von Möllendorff (1901b), Zilch (1953), Vermeulen and Maassen (2003)^1^	VN2019-003, VN2019-010, VN2019-018	Fig. 9F
Diplommatinidae	*Diplommatina tonkiniana* Jaeckel, 1950	Jaeckel (1950), Vermeulen and Maassen (2003)^5^	VN2019-008	Fig. 9J
Diplommatinidae	*Diplommatina* sp. vi-a2 *sensu* Vermeulen & Maassen 2003	Vermeulen and Maassen (2003) ^5^	VN2019-008, VN2019-011, VN2019-017	Fig. 9G
Diplommatinidae	*Diplommatina* sp. vi-a3 *sensu* Vermeulen & Maassen 2003	Vermeulen and Maassen (2003) ^5^	VN2019-010, VN2019-025, VN2019-026, VN2019-028	Fig. 9H
Diplommatinidae	*Diplommatina* sp. vi-a4 *sensu* Vermeulen & Maassen 2003	Vermeulen and Maassen (2003) ^5^	VN2019-003, VN2019-005	Fig. 9I
Pollicariidae	*Pollicaria rochebruni* (Mabille, 1887)	Mabille (1887b), Vermeulen and Maassen (2003)^1^, Kongim et al. (2013), Minton et al. (2017)	VN2019-008, VN2019-009, VN2019-010, VN2019-011, VN2019-017, VN2019-018, VN2019-022, VN2019-027, VN2019-030, VN2019-032, VN2019-035, VN2019-036	Fig. 8J, data package
Pupinidae	*Pseudopomatias amoenus* Möllendorff, 1885	von Möllendorff (1885), Vermeulen and Maassen (2003)^1^, Páll-Gergely et al. (2015a)	VN2019-025	Fig. 8K
Pupinidae	*Pupina anceyi* Bavay & Dautzenberg, 1899	Bavay and Dautzenberg (1899a), Vermeulen and Maassen (2003)^1^, Jirapatrasilp et al. (2022)	VN2019-005, VN2019-008, VN2019-018, VN2019-026^3^, VN2019-027, VN2019-036	Fig. 9A
Pupinidae	*Pupina dorri dorri* Dautzenberg, 1894	Dautzenberg (1894), Vermeulen and Maassen (2003)^1^, Jirapatrasilp et al. (2022)	VN2019-017	Fig. 9B
Pupinidae	*Pupina solidula* Möllendorff, 1901	von Möllendorff (1901a), Vermeulen and Maassen (2003)^1^, Jirapatrasilp et al. (2022)	VN2019-003, VN2019-008, VN2019-009, VN2019-010, VN2019-011, VN2019-017, VN2019-018, VN2019-019, VN2019-022, VN2019-025, VN2019-026, VN2019-027, VN2019-028, VN2019-035	Fig. 9C
Pupinidae	*Tylotoechus exclamationis* (Mabille, 1887)	Mabille (1887a), Vermeulen and Maassen (2003)^1^, Jirapatrasilp et al. (2022), Jirapatrasilp et al. (2025)	VN2019-015	Fig. 9D
Pupinidae	*Vargapupa oharai* Páll-Gergely, 2015	Vermeulen and Maassen (2003)^1^, Páll-Gergely et al. (2015a)	VN2019-010, VN2019-017, VN2019-035, VN2019-036	Fig. 8L
**Order Systellommatophora**				
Rathouisiidae	*Atopos* sp. A	Sarasin and Sarasin (1899) [generic assignment], Schilthuizen and Liew (2008) [generic assignment]	VN2019-025, VN2019-026^2^	Fig. 10A, data package (see also Fig. 4E)
Veronicellidae	gen. inc. sp. A	Schilthuizen and Liew (2008) [familial assignment]	VN2019-001	Fig. 9K
Veronicellidae	gen. inc. sp. B	Schilthuizen and Liew (2008) [familial assignment]	VN2019-003, VN2019-022	Fig. 9L, data package (see also Fig. 4F)
Veronicellidae	gen. inc. sp. C	Schilthuizen and Liew (2008) [familial assignment]	VN2019-023	Fig. 9M, data package
**Order Stylommatophora**				
Achatinidae	*Allopeas clavulinum* (Potiez & Michaud, 1838)	Potiez and Michaud (1838), Vermeulen and Maassen (2003)^1^, Vermeulen and Liew (2022)	VN2019-008, VN2019-035	Fig. 10B
Achatinidae	*Allopeas gracile* (Hutton, 1834)	Hutton (1834), Vermeulen and Maassen (2003)^1^, Vermeulen and Liew (2022)	VN2019-003^2^, VN2019-008^2^, VN2019-014^2^	Fig. 10C^6^
Achatinidae	*Curvella* sp. vi-1 *sensu* Vermeulen & Maassen 2003	Vermeulen and Maassen (2003) ^1^	VN2019-008	Fig. 10D
Achatinidae	*Paropeas achatinaceum* (Pfeiffer, 1846)	Pfeiffer (1846), Vermeulen and Maassen (2003)^7^, Vermeulen and Liew (2022)	VN2019-008^2,3^, VN2019-014	Fig. 10E
Achatinidae	*Prosopeas ventrosulum* Bavay & Dautzenberg, 1909	Bavay and Dautzenberg (1909)	VN2019-010^2^, VN2019-036	Fig. 10F
Ariophantidae	*Macrochlamys despecta* (Mabille, 1887)	Mabille (1887a), Mabille (1887b), Vermeulen and Maassen (2003)^1^, Pholyotha et al. (2020)	VN2019-002, VN2019-003^2^, VN2019-010, VN2019-014^2,3^, VN2019-017, VN2019-018, VN2019-024, VN2019-025, VN2019-026, VN2019-027, VN2019-028, VN2019-030, VN2019-035, VN2019-036^2^	Fig. 10J, Fig. 10K, data package
Ariophantidae	*Macrochlamys douvillei* Dautzenberg & Fischer, 1906	Dautzenberg and Fischer (1906), Vermeulen and Maassen (2003)^1^, Pholyotha et al. (2020)	VN2019-003, VN2019-008, VN2019-009^2,3^, VN2019-010, VN2019-011, VN2019-014, VN2019-015, VN2019-022, VN2019-025, VN2019-030, VN2019-035, VN2019-036	Fig. 10L, Fig. 10M, data package (see also Fig. 5A)
Ariophantidae	*Macrochlamys tenuigranosa* Dautzenberg, 1894	Dautzenberg (1894), Pholyotha et al. (2020)	VN2019-025^2^, VN2019-035	Fig. 10P
Ariophantidae	*Macrochlamys* sp. A	Schileyko (2003) [generic assignment]	VN2019-017, VN2019-024, VN2019-027^2^	Fig. 10H, Fig. 10I
Ariophantidae	*Macrochlamys* sp. vi-2 *sensu* Vermeulen & Maassen 2003	Vermeulen and Maassen (2003) ^1^	VN2019-010, VN2019-017, VN2019-025, VN2019-026, VN2019-035	Fig. 10N, Fig. 10O
Ariophantidae	*Macrochlamys* sp. vi-3 *sensu* Vermeulen & Maassen 2003	Vermeulen and Maassen (2003) ^1^	VN2019-003, VN2019-022	Fig. 10G, data package (see also Fig. 5B)
Ariophantidae	*Megaustenia fragile* (Möllendorff, 1901)	von Möllendorff (1901a), Vermeulen and Maassen (2003)^1^, Schileyko (2011)	VN2019-003, VN2019-005^2^, VN2019-010, VN2019-011, VN2019-019^2^, VN2019-024, VN2019-025, VN2019-026, VN2019-027, VN2019-028, VN2019-035, VN2019-036	Fig. 11A, Fig. 11B, data package
Ariophantidae	*Megaustenia messageri* (Bavay & Dautzenberg, 1909)	Bavay and Dautzenberg (1909), Vermeulen and Maassen (2003)^1^, Schileyko (2011)	VN2019-022	Fig. 11C, data package (see also Fig. 5C)
Ariophantidae	*Microcystina* sp. A	Schileyko (2003) [generic assignment], Vermeulen and Liew (2022) [generic assignment]	VN2019-014, VN2019-015	Fig. 11E
Ariophantidae	*Microcystina* sp. B	Vermeulen and Maassen (2003) ^1^	VN2019-010	Fig. 11F
Ariophantidae	*Microcystina* sp. C	Schileyko (2003) [generic assignment], Vermeulen and Liew (2022) [generic assignment]	VN2019-011	Fig. 11G, data package
Ariophantidae	*Microcystina* sp. D	Schileyko (2003) [generic assignment], Vermeulen and Maassen (2003)^1^, Vermeulen and Liew (2022) [generic assignment]	VN2019-008, VN2019-025	Fig. 11H
Ariophantidae	*Microcystina* sp. E	Vermeulen and Maassen (2003) ^1^	VN2019-017, VN2019-018, VN2019-025, VN2019-035, VN2019-036	Fig. 11I
Ariophantidae	*Microcystina* sp. vi-b01 *sensu* Vermeulen & Maassen 2003	Vermeulen and Maassen (2003) ^1^	VN2019-003	Fig. 11J
Ariophantidae	*Microcystina* sp. vi-b03 *sensu* Vermeulen & Maassen 2003	Vermeulen and Maassen (2003) ^1^	VN2019-010, VN2019-024^2^, VN2019-025	Fig. 11K
Ariophantidae	*Microcystina* sp. vi-b06 *sensu* Vermeulen & Maassen 2003	Vermeulen and Maassen (2003) ^1^	VN2019-003, VN2019-008, VN2019-011	Fig. 11L
Ariophantidae	*Microcystina* sp. vi-b10 *sensu* Vermeulen & Maassen 2003	Vermeulen and Maassen (2003) ^1^	VN2019-017, VN2019-024^2,3^	Fig. 11M^6^
Ariophantidae	*Parmarion* sp. A	Simroth (1894) [generic assignment], Schilthuizen and Liew (2008) [generic assignment]	VN2019-018^2^, VN2019-032	Fig. 11D, data package^4^ (see also Fig. 5D)
Camaenidae	*Aegista coudeini* (Bavay & Dautzenberg, 1900)	Bavay and Dautzenberg (1900a), Bavay and Dautzenberg (1900b), Vermeulen and Maassen (2003)^1^, Inkhavilay et al. (2019)	VN2019-006, VN2019-008, VN2019-014, VN2019-015^2^, VN2019-018, VN2019-019, VN2019-022, VN2019-024, VN2019-025, VN2019-026, VN2019-032	Fig. 12L, data package
Camaenidae	*Aegista pseudotrochula* (Bavay & Dautzenberg, 1909)	Bavay and Dautzenberg (1909), Vermeulen and Maassen (2003)^1^, Inkhavilay et al. (2019)	VN2019-003, VN2019-005, VN2019-008, VN2019-035, VN2019-036	Fig. 12M
Camaenidae	*Amphidromus roseolabiatus* Fulton, 1896	see Do et al. (2024)	VN2019-009, VN2019-017, VN2019-018^2^, VN2019-020, VN2019-027^2^, VN2019-032, VN2019-035^2^, VN2019-036	Fig. 13J, data package (see also Fig. 6A)
Camaenidae	*Bradybaena jourdyi* (Morlet, 1886)^8^	Morlet (1886a), Inkhavilay et al. (2019)	VN2019-005^2,3^	data package^4,9^
Camaenidae	*Camaena billeti* (Fischer, 1898)	Fischer (1898), Vermeulen and Maassen (2003)^1^, Schileyko (2011)	VN2019-009^2,3^, VN2019-010, VN2019-017, VN2019-018, VN2019-024^2,3^, VN2019-025, VN2019-026^2^, VN2019-028	Fig. 13A, data package^4^
Camaenidae	*Camaena choboensis* (Mabille, 1889)	Mabille (1889), Vermeulen and Maassen (2003)^1^, Inkhavilay et al. (2019)	VN2019-008, VN2019-014, VN2019-017^2^, VN2019-029^2^	Fig. 13B, data package^4^
Camaenidae	*Camaena chuongi* Thach, 2016	Vermeulen and Maassen (2003)^1^, Nguyen (2016a)	VN2019-003, VN2019-005, VN2019-008, VN2019-017, VN2019-018, VN2019-022^2^, VN2019-024, VN2019-025, VN2019-026^2^, VN2019-027, VN2019-028, VN2019-032^2^, VN2019-034^2^	Fig. 13C, data package
Camaenidae	*Camaena onae* Thach, 2016	Vermeulen and Maassen (2003)^1^, Nguyen (2016b)	VN2019-008^2,3^, VN2019-009, VN2019-010, VN2019-011, VN2019-017^2,3^, VN2019-021^2^, VN2019-025^2^, VN2019-026^2^, VN2019-027, VN2019-028, VN2019-035, VN2019-036	Fig. 13D
Camaenidae	*Camaena vanbuensis* Smith, 1896	Smith (1896), Vermeulen and Maassen (2003)^1^, Inkhavilay et al. (2019)	VN2019-010, VN2019-011, VN2019-017, VN2019-024, VN2019-025, VN2019-026, VN2019-027	Fig. 13F
Camaenidae	*Camaena* sp. A	Vermeulen and Maassen (2003)^1^, Sutcharit and Panha (2021) [generic assignment]	VN2019-005, VN2019-009^2,3^, VN2019-013^2^, VN2019-014^2^, VN2019-027, VN2019-035, VN2019-036	Fig. 13E
Camaenidae	*Chloritis* sp. A	Sutcharit and Panha (2010) [generic assignment]	VN2019-022, VN2019-024, VN2019-028	Fig. 13G, data package (see also Fig. 6B)
Camaenidae	*Ganesella oxytropis* (Möllendorff, 1901)	von Möllendorff (1901b), Zilch (1966), Vermeulen and Maassen (2003)^1^	VN2019-003, VN2019-005, VN2019-008^2,3^, VN2019-009, VN2019-010, VN2019-011^2,3^, VN2019-012, VN2019-017, VN2019-026, VN2019-027, VN2019-028, VN2019-033^2^, VN2019-035, VN2019-036	Fig. 12N, data package
Camaenidae	*Ganesella procera* Gude, 1902	Gude (1902), Gude (1903)	VN2019-005	Fig. 12O, data package
Camaenidae	*Globotrochus onestera* (Mabille, 1887)	Mabille (1887b), Sutcharit et al. (2019)	VN2019-017, VN2019-029, VN2019-032	Fig. 13H
Camaenidae	*Neocepolis merarcha* (Mabille, 1888)	Mabille (1888), Vermeulen and Maassen (2003)^1^, Sutcharit et al. (2007)	VN2019-025, VN2019-026, VN2019-027, VN2019-028	Fig. 13I, data package (see also Fig. 6C)
Camaenidae	*Pseudobuliminus franzhuberi* Thach, 2018	Vermeulen and Maassen (2003)^1^, Nguyen (2018)	VN2019-003, VN2019-005, VN2019-009, VN2019-011, VN2019-022, VN2019-025, VN2019-026, VN2019-028	Fig. 13K, data package (see also Fig. 6D)
Chronidae	*Kaliella haiphongensis* Dautzenberg, 1894	Dautzenberg (1894), Vermeulen and Maassen (2003)^7^, Schileyko (2011)	VN2019-003^2,3^, VN2019-014, VN2019-015	Fig. 12A
Chronidae	*Kaliella joubini* Dautzenberg & Fischer, 1905	Dautzenberg and Fischer (1905), Vermeulen and Maassen (2003)^1^, Schileyko (2011)	VN2019-009, VN2019-024, VN2019-036	Fig. 12B
Chronidae	*Kaliella subelongata* Bavay & Dautzenberg, 1912	Bavay and Dautzenberg (1912), Vermeulen and Maassen (2003)^1^, Schileyko (2011)	VN2019-003^2^	Fig. 12K^6^
Chronidae	*Kaliella tongkingensis* Möllendorff, 1901	von Möllendorff (1901a), Vermeulen and Maassen (2003)^1^, Schileyko (2011)	VN2019-018	Fig. 12J^6^
Chronidae	*Kaliella* sp. A	Vermeulen et al. (2015) [generic assignment]	VN2019-008, VN2019-032	Fig. 12C
Chronidae	*Kaliella* sp. B	Vermeulen et al. (2015) [generic assignment]	VN2019-005, VN2019-008, VN2019-014	Fig. 12D
Chronidae	*Kaliella* sp. C	Vermeulen et al. (2015) [generic assignment]	VN2019-003^2,3^, VN2019-008^2,3^, VN2019-032	Fig. 12E
Chronidae	*Kaliella* sp. D	Vermeulen et al. (2015) [generic assignment]	VN2019-018^2^, VN2019-028	Fig. 12F
Chronidae	*Kaliella* sp. E	Vermeulen et al. (2015) [generic assignment]	VN2019-014	Fig. 12G
Chronidae	*Kaliella* sp. F^8^	Vermeulen and Maassen (2003)^1^, Vermeulen et al. (2015) [generic assignment]	VN2019-005^2,3^	Fig. 12H^6^
Chronidae	*Kaliella* sp. G	Vermeulen and Maassen (2003)^1^, Vermeulen et al. (2015) [generic assignment]	VN2019-025^2^	Fig. 12I^6^
Clausiliidae	*Euryauchenia fischeri reticulata* (Nordsieck, 2002)	Nordsieck (2002), Nordsieck (2011)	VN2019-010, VN2019-011, VN2019-017	Fig. 14A
Clausiliidae	*Megalauchenia proctostoma forceps* (Loosjes & Loosjes-van Bemmel, 1973)	Loosjes and Loosjes-van Bemmel (1973), Vermeulen and Maassen (2003)^1^, Nordsieck (2011)	VN2019-003, VN2019-005, VN2019-006, VN2019-008, VN2019-009, VN2019-010, VN2019-017, VN2019-018, VN2019-024, VN2019-025, VN2019-026, VN2019-027, VN2019-028, VN2019-032, VN2019-035, VN2019-036	Fig. 14B
Clausiliidae	*Megalauchenia proctostoma proctostoma* (Mabille, 1889)	Mabille (1889), Loosjes and Loosjes-van Bemmel (1973), Nordsieck (2011)	VN2019-014	Fig. 14C
Clausiliidae	*Oospira abstrusa abstrusa* (Szekeres, 1970)	Szekeres (1970), Grego et al. (2014), Nordsieck (2021)	VN2019-013, VN2019-014, VN2019-036	Fig. 14D
Clausiliidae	*Oospira eregia* (Szekeres, 1969)	Szekeres (1969), Nordsieck (2016)	VN2019-010, VN2019-035	Fig. 14E
Clausiliidae	*Oospira miranda* (Loosjes & Loosjes-van Bemmel, 1973)	Loosjes and Loosjes-van Bemmel (1973), Vermeulen and Maassen (2003)^1^, Nordsieck (2011)	VN2019-003, VN2019-005, VN2019-008, VN2019-009, VN2019-010, VN2019-011, VN2019-015, VN2019-025, VN2019-026, VN2019-027, VN2019-028, VN2019-035, VN2019-036	Fig. 14F, data package (see also Fig. 6E)
Clausiliidae	*Oospira vanbuensis* (Bavay & Dautzenberg, 1899)	Bavay and Dautzenberg (1899a), Szekeres (1970), Vermeulen and Maassen (2003)^1^, Nordsieck (2011), Nordsieck (2021)	VN2019-003, VN2019-008, VN2019-013, VN2019-017, VN2019-018, VN2019-022, VN2019-025, VN2019-035, VN2019-036	Fig. 14G
Clausiliidae	*Phaedusa dichroa* (Bavay & Dautzenberg, 1899)	Bavay and Dautzenberg (1899b), Bavay and Dautzenberg (1915), Nordsieck (2011)	VN2019-013, VN2019-014, VN2019-018, VN2019-036	Fig. 14H, data package
Clausiliidae	*Phaedusa lypra* (Mabille, 1887)	Mabille (1887a), Vermeulen and Maassen (2003)^1^, Nordsieck (2011), Nordsieck (2021)	VN2019-008, VN2019-014, VN2019-036	Fig. 14I
Clausiliidae	*Phaedusa stenothyra* Möllendorff, 1901	von Möllendorff (1901b), Zilch (1954)	VN2019-014, VN2019-015, VN2019-035, VN2019-036	Fig. 14J
Diapheridae	*Sinoennea* sp. A	Páll-Gergely et al. (2020b) [generic assignment]	VN2019-014^2^	n.a.
Dyakiidae	*Quantula* sp. 3 *sensu* Jirapatrasilp et al. 2021	Vermeulen and Maassen (2003)^1^, Jirapatrasilp et al. (2021)	VN2019-005, VN2019-008, VN2019-009, VN2019-010, VN2019-014, VN2019-017, VN2019-025^2^, VN2019-027, VN2019-028, VN2019-035, VN2019-036	Fig. 14L, data package (see also Fig. 5E)
Enidae	*Apoecus messageri* (Bavay & Dautzenberg, 1900)	Bavay and Dautzenberg (1900a), Vermeulen and Maassen (2003)^1^, Schileyko (2011), Köhler et al. (2017)	VN2019-003, VN2019-005	Fig. 13L, data package
Helicarionidae	*Chalepotaxis infantilis* (Gredler, 1881)	Gredler (1881), Vermeulen and Maassen (2003)^1^, Páll-Gergely et al. (2017a)	VN2019-009^2^, VN2019-014, VN2019-024	Fig. 15C
Helicarionidae	*Durgella* sp. A	Solem (1966) [generic assignment], Schileyko (2002) [generic assignment], Vermeulen and Maassen (2003)^1^	VN2019-008, VN2019-014^2,3^	Fig. 15A
Helicarionidae	*Durgella* sp. B	Solem (1966) [generic assignment], Schileyko (2002) [generic assignment]	VN2019-031	Fig. 15B, data package
Helicarionidae	*Rahula jucunda* (Bavay & Dautzenberg, 1912)	Bavay and Dautzenberg (1912), Vermeulen and Maassen (2003)^1^, Gittenberger et al. (2017)	VN2019-028	Fig. 15D
Helicarionidae	*Sesara* sp. vi-01 *sensu* Vermeulen & Maassen 2003	Vermeulen and Maassen (2003) ^1^	VN2019-003^2^	Fig. 15G^6^
Helicarionidae	*Sitala confinis* (Möllendorff, 1901)	von Möllendorff (1901a), Vermeulen and Maassen (2003)^1^, Schileyko (2011)	VN2019-003	Fig. 15E
Helicarionidae	*Teraia contempta* (Bavay & Dautzenberg, 1909)	Bavay and Dautzenberg (1909), Schileyko (2011)	VN2019-024	Fig. 15F
Hypselostomatidae	*Bensonella* sp. A^8^	Vermeulen and Maassen (2003)^1^, Panha and Burch (2005) [generic assignment], Páll-Gergely and White (2022) [generic assignment], Gojšina et al. (2025) [generic assignment]	VN2019-008^2,3^	Fig. 15H^6^
Philomycidae	*Meghimatium pictum* (Stoliczka, 1873)	Stoliczka (1873), Hoffmann (1924), Wiktor et al. (2000), Schilthuizen and Liew (2008)	VN2019-010, VN2019-024, VN2019-027^2^, VN2019-030	Fig. 14K, data package (see also Fig. 6F)
Plectopylidae	*Gudeodiscus messageri raheemi* Páll-Gergely & Hunyadi, 2015	Vermeulen and Maassen (2003)^1^, Páll-Gergely et al. (2015b)	VN2019-010, VN2019-017, VN2019-025, VN2019-035, VN2019-036^2,3^	Fig. 14M
Plectopylidae	*Gudeodiscus phlyarius* (Mabille, 1887)	Mabille (1887b), Páll-Gergely et al. (2015b)	VN2019-025	Fig. 14N
Streptaxidae	*Haploptychius diespiter* (Mabille, 1887)	Mabille (1887a), Mabille (1887b), Vermeulen and Maassen (2003)^1^, Inkhavilay et al. (2016)	VN2019-003, VN2019-008, VN2019-009, VN2019-010, VN2019-014, VN2019-025, VN2019-028, VN2019-035, VN2019-036	Fig. 15I, data package
Streptaxidae	*Perrottetia* sp. A	Siriboon et al. (2013) [generic assignment], Do (2017) [generic assignment]	VN2019-008	Fig. 15J
Trochomorphidae	*Trochomorpha paviei* (Morlet, 1885)	Morlet (1885), Vermeulen and Maassen (2003)^1^, Inkhavilay et al. (2023)	VN2019-003, VN2019-008, VN2019-009, VN2019-011^2^, VN2019-014, VN2019-018, VN2019-024, VN2019-025, VN2019-027, VN2019-035, VN2019-036	Fig. 15K, data package (see also Fig. 5F)
Trochomorphidae	*Trochomorpha saigonensis* (Crosse, 1867)	Crosse (1867), Inkhavilay et al. (2023)	VN2019-003^3^, VN2019-005, VN2019-008, VN2019-013, VN2019-014	Fig. 15L, data package^4,9^
Trochomorphidae	*Trochomorpha* sp. A	Vermeulen and Maassen (2003)^1^, Inkhavilay et al. (2023) [generic assignment]	VN2019-014^3^, VN2019-018, VN2019-022, VN2019-026	Fig. 15M
Valloniidae	*Pupisoma dioscoricola* (Adams, 1845)	Adams (1845), Vermeulen and Maassen (2003)^1^, Hausdorf (2007)	VN2019-014	Fig. 15N

^1^Compared with material from JJ Vermeulen’s set of the 1998 Cuc Phuong National Park collection. ^2^All specimens clearly identified as juvenile. ^3^All specimens with uncertain identification. ^4^Only photographs of material clearly identified as juvenile in the data package. ^5^Based on unpublished data provided by JJ Vermeulen. ^6^Material from JJ Vermeulen’s set of the 1998 Cuc Phuong National Park collection shown. ^7^Compared with non-Cuc Phuong National Park material from Vermeulen and Maassen (2003) provided by JJ Vermeulen. ^8^Only material with uncertain identification recorded. ^9^Only photographs of material with uncertain identification in the data package.

**Table 4.** List of terrestrial molluscs currently known from Cuc Phuong National Park, including family and (sub-)species name, literature references concerning the respective taxon in Cuc Phuong National Park, and recording occasions considered for our mark-recapture-based species richness estimation of shelled land molluscs. This list includes both, taxa collected during the 2019 VIETBIO inventory work and taxa plausibly reported in the literature (no collection material of taxa that were not found in 2019 was evaluated). As the reports of Vermeulen and Whitten (1998) and Vermeulen and Maassen (2003) were based on the same Cuc Phuong National Park material, we only took the latter into account, as it contains the more thorough species identification. Recording occasion 1 refers to the 1998 collection, and recording occasion 2 to our 2019 collection.

**Family**	**(Sub-)Species**	**Literature references concerning Cuc Phuong National Park**	**Recording occasions considered for species richness estimation**
**Order Cycloneritida**			
Helicinidae	*Aphanoconia hungerfordiana tonkinensis* Möllendorff, 1909	this study	2
Helicinidae	*Geotrochatella insignis* Dautzenberg, 1896	Vermeulen and Maassen (2003) [*Geotrochatella mouhoti*], Yamazaki et al. (2007) [*Calybium* sp.]^1^, this study	1, 2
Hydrocenidae	*Georissa chrysacme* Möllendorff, 1900	Vermeulen and Maassen (2003)	1
Hydrocenidae	*Georissa* sp. vi-2 *sensu* Vermeulen & Maassen 2003	Vermeulen and Maassen (2003)	1
Hydrocenidae	*Georissa* sp. vi-4 *sensu* Vermeulen & Maassen 2003	Vermeulen and Maassen (2003), this study	1, 2
**Order Architaenioglossa**			
Cyclophoridae	*Cyclophorus cucphuongensis* Oheimb, 2019	Vermeulen and Maassen (2003) [*Cyclophorus volvulus*], Yamazaki et al. (2007) [*Cyclophorus* sp. 1]^1^, Raheem et al. (2018) [*Cyclophorus* sp. (NHMUK 20170132)]^2^, von Oheimb et al. (2018) [lineage 10], von Oheimb et al. (2019), this study	1, 2
Cyclophoridae	*Cyclophorus paracucphuongensis* Oheimb, 2019	von Oheimb et al. (2019), this study	2
Cyclophoridae	*Cyclophorus takumisaitoi* Hirano, 2019	von Oheimb et al. (2019)	
Cyclophoridae	*Cyclophorus* sp. (lineage 01 *sensu* Oheimb et al. 2018)	Vermeulen and Maassen (2003) [*Cyclophorus malayanus*], Nantarat et al. (2014) [*Cyclophorus songmaensis*], von Oheimb et al. (2018), this study	1, 2
Cyclophoridae	*Cyclophorus* sp. (lineage 08 *sensu* Oheimb et al. 2018)	Vermeulen and Maassen (2003) [*Cyclophorus cambodgensis*], Yamazaki et al. (2007) [*Cyclophorus* sp. 2], Ivanova et al. (2016) [*Cyclophorus* sp.]^3^, Raheem et al. (2018) [*Cyclophorus* sp. (NHMUK 20170133)]^2^, von Oheimb et al. (2018), von Oheimb et al. (2019) [*Cyclophorus* sp. (mutual group 39)], this study	1, 2
Cyclophoridae	*Cyclotus danieli* (Morlet, 1886)	Vermeulen and Maassen (2003) [*Pterocyclos danieli*, *Pterocyclos fischerianus*^4^], this study	1, 2
Cyclophoridae	*Dicharax depressus* (Bavay & Dautzenberg, 1912)	Vermeulen and Maassen (2003) [*Chamalycaeus heudei*], Yamazaki et al. (2007) [*Chamalycaeus* sp.], this study	1, 2
Cyclophoridae	*Dicharax fimbriatus* (Bavay & Dautzenberg, 1912)^5^	Vermeulen and Maassen (2003) [*Chamalycaeus fimbriatus*]	1
Cyclophoridae	*Dicharax fraterculus* (Bavay & Dautzenberg, 1900)^5^	Vermeulen and Maassen (2003) [*Chamalycaeus fraterculus*]	1
Cyclophoridae	*Dioryx dongiensis* Varga, 1972	Varga (1972), Vermeulen and Maassen (2003), Páll-Gergely et al. (2020c), this study	1, 2
Cyclophoridae	*Dioryx messageri* (Bavay & Dautzenberg, 1900)	Vermeulen and Maassen (2003), Yamazaki et al. (2007), Páll-Gergely et al. 2020c, this study	1, 2
Cyclophoridae	*Dioryx pocsi* Varga, 1972	Varga (1972), Vermeulen and Maassen (2003), Páll-Gergely et al. (2020c)	1
Cyclophoridae	*Japonia* sp. vi-06 *sensu* Vermeulen & Maassen 2003^6^	Vermeulen and Maassen (2003)	1
Cyclophoridae	*Lagocheilus hypselospirus* Möllendorff, 1901	Vermeulen and Maassen (2003) [*Japonia hypselospira*], this study	1, 2
Cyclophoridae	*Lagocheilus* sp. A	Vermeulen and Maassen (2003) [*Japonia* sp. vi-01], this study	1, 2
Cyclophoridae	*Lagocheilus* sp. B	Vermeulen and Maassen (2003) [*Japonia* sp. vi-04], this study	1, 2
Cyclophoridae	*Lagocheilus* sp. C	Vermeulen and Maassen (2003) [*Japonia* sp. vi-05], this study	1, 2
Cyclophoridae	*Metalycaeus heudei* (Bavay & Dautzenberg, 1900)	Varga (1974) [Chamalycaeus (Dicharax) fractus], Vermeulen and Maassen (2003) [*Chamalycaeus fractus*], Páll-Gergely et al. (2017b)	1
Cyclophoridae	*Pincerna mouhoti* (Pfeiffer, 1863)	Vermeulen and Maassen (2003) [*Alycaeus vanbuensis*], Páll-Gergely (2023), this study	1, 2
Cyclophoridae	*Platyrhaphe leucacme* Möllendorff, 1901	Vermeulen and Maassen (2003) [*Platyraphe leucacme* (sic)]	1
Cyclophoridae	*Rhiostoma morleti* Dautzenberg & Fischer, 1906	this study	2
Cyclophoridae	*Scabrina* sp. A	Vermeulen and Maassen (2003) [*Scabrina hirsuta*], Yamazaki et al. (2007) [*Scabrina locardi*], this study	1, 2
Cyclophoridae	*Scabrina* sp. B	Vermeulen and Maassen (2003) [*Pterocyclos* sp. vi-01], this study	1, 2
Cyclophoridae	*Scabrina* sp. vi-01 *sensu* Vermeulen & Maassen 2003	Vermeulen and Maassen (2003), this study	1, 2
Diplommatinidae	*Diplommatina debilis* Bavay & Dautzenberg, 1904	Vermeulen and Maassen (2003), this study	1, 2
Diplommatinidae	*Diplommatina demangei* Bavay & Dautzenberg, 1912^7^	Vermeulen and Maassen (2003), Yamazaki et al. (2007) [*Diplommatina* sp. 2]^8^	1
Diplommatinidae	*Diplommatina herziana* Möllendorff, 1886	Vermeulen and Maassen (2003)	1
Diplommatinidae	*Diplommatina scolops* Möllendorff, 1901	Vermeulen and Maassen (2003), Yamazaki et al. (2007) [*Diplommatina balansai*], this study	1, 2
Diplommatinidae	*Diplommatina tonkiniana* Jaeckel, 1950	Vermeulen and Maassen (2003) [*Diplommatina* sp. vi-a1], this study	1, 2
Diplommatinidae	*Diplommatina* sp. vi-a2 *sensu* Vermeulen & Maassen 2003	Vermeulen and Maassen (2003), this study	1, 2
Diplommatinidae	*Diplommatina* sp. vi-a3 *sensu* Vermeulen & Maassen 2003	Vermeulen and Maassen (2003), this study	1, 2
Diplommatinidae	*Diplommatina* sp. vi-a4 *sensu* Vermeulen & Maassen 2003	Vermeulen and Maassen (2003), Yamazaki et al. (2007) [*Diplommatina* sp. 1], this study	1, 2
Diplommatinidae	*Diplommatina* sp. vi-r1 *sensu* Vermeulen & Maassen 2003	Vermeulen and Maassen (2003)	1
Diplommatinidae	*Diplommatina* sp. vi-sp1 *sensu* Vermeulen & Maassen 2003	Vermeulen and Maassen (2003)	1
Pollicariidae	*Pollicaria rochebruni* (Mabille, 1887)	Vermeulen and Maassen (2003) [*Pollicaria gravida*], Kongim et al. (2010) [*Pollicaria gravida*], Kongim et al. (2013) [*Pollicaria crossei*, *Pollicaria rochebruni*], Minton et al. (2017), Jirapatrasilp et al. (2025), this study	1, 2
Pupinidae	*Pseudopomatias amoenus* Möllendorff, 1885	Vermeulen and Maassen (2003) [*Pseudopomatias fulvus*], Páll-Gergely et al. (2015a), this study	1, 2
Pupinidae	*Pupina anceyi* Bavay & Dautzenberg, 1899	Vermeulen and Maassen (2003) [*Pupina brachysoma*], Yamazaki et al. (2007) [*Pupina* sp.], this study	1, 2
Pupinidae	*Pupina dorri dorri* Dautzenberg, 1894	Vermeulen and Maassen (2003) [*Pupina dorri*], this study	1, 2
Pupinidae	*Pupina solidula* Möllendorff, 1901	Vermeulen and Maassen (2003) [*Pupina flava*], this study	1, 2
Pupinidae	*Tylotoechus exclamationis* (Mabille, 1887)	Vermeulen and Maassen (2003) [*Pupina exclamationis*], this study	1, 2
Pupinidae	*Vargapupa oharai* Páll-Gergely, 2015	Vermeulen and Maassen (2003) [*Pseudopomatias* sp.], Páll-Gergely et al. (2015a), this study	1, 2
**Order Littorinimorpha**			
Assimineidae	*Acmella* sp. 4 *sensu* Vermeulen & Maassen 2003	Vermeulen and Maassen (2003)	1
**Order Ellobiida**			
Ellobiidae	*Carychium javanum* Möllendorff, 1897	Vermeulen and Maassen (2003)	1
**Order Systellommatophora**			
Rathouisiidae	*Atopos* sp. A	this study	
Veronicellidae	gen. inc. sp. A	this study	
Veronicellidae	gen. inc. sp. B	this study	
Veronicellidae	gen. inc. sp. C	this study	
**Order Stylommatophora**			
Achatinidae	*Allopeas clavulinum* (Potiez & Michaud, 1838)	Vermeulen and Maassen (2003) [*Lamellaxis clavulinus*], this study	1, 2
Achatinidae	*Allopeas gracile* (Hutton, 1834)	Vermeulen and Maassen (2003) [*Lamellaxis gracilis*], this study	1, 2
Achatinidae	*Curvella tonkiniana* Jaeckel, 1950	Vermeulen and Maassen (2003)	1
Achatinidae	*Curvella* sp. vi-1 *sensu* Vermeulen & Maassen 2003	Vermeulen and Maassen (2003), this study	1, 2
Achatinidae	*Glessula paviei* Morlet, 1893	Vermeulen and Maassen (2003)	1
Achatinidae	*Lissachatina fulica* (Bowdich, 1822)^9^	Fontanilla et al. (2014) [*Achatina fulica*]	
Achatinidae	*Paropeas achatinaceum* (Pfeiffer, 1846)	Vermeulen and Maassen (2003), this study	1, 2
Achatinidae	*Prosopeas ventrosulum* Bavay & Dautzenberg, 1909	this study	2
Ariophantidae	*Macrochlamys despecta* (Mabille, 1887)	Vermeulen and Maassen (2003), this study	1, 2
Ariophantidae	*Macrochlamys douvillei* Dautzenberg & Fischer, 1906	Vermeulen and Maassen (2003), this study	1, 2
Ariophantidae	*Macrochlamys tenuigranosa* Dautzenberg, 1894	this study	2
Ariophantidae	*Macrochlamys* sp. A	this study	2
Ariophantidae	*Macrochlamys* sp. vi-2 *sensu* Vermeulen & Maassen 2003	Vermeulen and Maassen (2003), this study	1, 2
Ariophantidae	*Macrochlamys* sp. vi-3 *sensu* Vermeulen & Maassen 2003	Vermeulen and Maassen (2003), this study	1, 2
Ariophantidae	*Megaustenia fragile* (Möllendorff, 1901)	Vermeulen and Maassen (2003) [*Megaustenia fragilis*, *Megaustenia imperator*], this study	1, 2
Ariophantidae	*Megaustenia messageri* (Bavay & Dautzenberg, 1909)	Vermeulen and Maassen (2003) [*Austenia messageri*], this study	1, 2
Ariophantidae	*Microcystina sinica* Möllendorff, 1885	Vermeulen and Maassen (2003)	1
Ariophantidae	*Microcystina* sp. A	this study	2
Ariophantidae	*Microcystina* sp. B	Vermeulen and Maassen (2003) [*Microcystina* sp. vi-b04 (partly)], this study	1, 2
Ariophantidae	*Microcystina* sp. C	this study	2
Ariophantidae	*Microcystina* sp. D	Vermeulen and Maassen (2003) [*Liardetia* sp. vi-r8], this study	1, 2
Ariophantidae	*Microcystina* sp. E	Vermeulen and Maassen (2003) [*Microcystina* sp. vi-b04 (partly)], this study	1, 2
Ariophantidae	*Microcystina* sp. vi-b01 *sensu* Vermeulen & Maassen 2003	Vermeulen and Maassen (2003), this study	1, 2
Ariophantidae	*Microcystina* sp. vi-b02 *sensu* Vermeulen & Maassen 2003	Vermeulen and Maassen (2003)	1
Ariophantidae	*Microcystina* sp. vi-b03 *sensu* Vermeulen & Maassen 2003	Vermeulen and Maassen (2003), this study	1, 2
Ariophantidae	*Microcystina* sp. vi-b05 *sensu* Vermeulen & Maassen 2003	Vermeulen and Maassen (2003)	1
Ariophantidae	*Microcystina* sp. vi-b06 *sensu* Vermeulen & Maassen 2003	Vermeulen and Maassen (2003), this study	1, 2
Ariophantidae	*Microcystina* sp. vi-b07 *sensu* Vermeulen & Maassen 2003	Vermeulen and Maassen (2003)	1
Ariophantidae	*Microcystina* sp. vi-b10 *sensu* Vermeulen & Maassen 2003	Vermeulen and Maassen (2003), this study	1, 2
Ariophantidae	*Microcystina* sp. vi-w1 *sensu* Vermeulen & Maassen 2003	Vermeulen and Maassen (2003)	1
Ariophantidae	*Microcystina* sp. vi-w3 *sensu* Vermeulen & Maassen 2003	Vermeulen and Maassen (2003)	1
Ariophantidae	*Parmarion* sp. A	this study	
Camaenidae	*Aegista coudeini* (Bavay & Dautzenberg, 1900)	Vermeulen and Maassen (2003) [*Ganesella coudeini*], this study	1, 2
Camaenidae	*Aegista pseudotrochula* (Bavay & Dautzenberg, 1909)	Vermeulen and Maassen (2003) [*Landouria ptychostyla*], this study	1, 2
Camaenidae	*Amphidromus roseolabiatus* Fulton, 1896	Do et al. (2024), this study	2
Camaenidae	*Bradybaena jourdyi* (Morlet, 1886)	this study^10^	2
Camaenidae	*Camaena billeti* (Fischer, 1898)	Vermeulen and Maassen (2003), this study	1, 2
Camaenidae	*Camaena choboensis* (Mabille, 1889)	Vermeulen and Maassen (2003) [*Camaena vayssierei*], this study	1, 2
Camaenidae	*Camaena chuongi* Thach, 2016	Vermeulen and Maassen (2003) [*Camaena gabrielae* (sic)], this study	1, 2
Camaenidae	*Camaena onae* Thach, 2016	Vermeulen and Maassen (2003) [*Camaena duporti*], this study	1, 2
Camaenidae	*Camaena vanbuensis* Smith, 1896	Vermeulen and Maassen (2003) [*Nesiohelix vorvonga*], this study	1, 2
Camaenidae	*Camaena* sp. A	Vermeulen and Maassen (2003) [*Chloritis* sp. vi-1], this study	1, 2
Camaenidae	*Chloritis* sp. A	this study	2
Camaenidae	*Ganesella oxytropis* (Möllendorff, 1901)	Vermeulen and Maassen (2003) [*Ganesella oxytropis*, *Ganesella fulvescens*], this study	1, 2
Camaenidae	*Ganesella procera* Gude, 1902	this study	2
Camaenidae	*Globotrochus onestera* (Mabille, 1887)	Sutcharit et al. (2019), this study	2
Camaenidae	*Neocepolis merarcha* (Mabille, 1888)	Vermeulen and Maassen (2003), Sutcharit et al. (2007), this study	1, 2
Camaenidae	*Neocepolis morleti* (Dautzenberg & d’Hamonville, 1887)	Sutcharit et al. (2007)	
Camaenidae	*Pseudobuliminus franzhuberi* Thach, 2018	Vermeulen and Maassen (2003) [*Pseudobuliminus* sp. vi-01], this study	1, 2
Chronidae	*Kaliella haiphongensis* Dautzenberg, 1894	Vermeulen and Maassen (2003) [*Liardetia haiphongensis*], this study	1, 2
Chronidae	*Kaliella joubini* Dautzenberg & Fischer, 1905	Vermeulen and Maassen (2003) [*Liardetia* sp. vi-k5], this study	1, 2
Chronidae	*Kaliella scandens* (Cox, 1872)	Vermeulen and Maassen (2003)	1
Chronidae	*Kaliella subelongata* Bavay & Dautzenberg, 1912	Vermeulen and Maassen (2003) [*Liardetia subelongata*], this study	1, 2
Chronidae	*Kaliella tongkingensis* Möllendorff, 1901	Vermeulen and Maassen (2003) [*Liardetia tonkingensis*], this study	1, 2
Chronidae	*Kaliella* sp. A	this study	2
Chronidae	*Kaliella* sp. B	this study	2
Chronidae	*Kaliella* sp. C	this study	2
Chronidae	*Kaliella* sp. D	this study	2
Chronidae	*Kaliella* sp. E	this study	2
Chronidae	*Kaliella* sp. F	Vermeulen and Maassen (2003) [*Liardetia* sp. vi-k1], this study^10^	1, 2
Chronidae	*Kaliella* sp. G	Vermeulen and Maassen (2003) [*Liardetia* sp. vi-r2], this study	1, 2
Clausiliidae	*Euryauchenia fischeri reticulata* (Nordsieck, 2002)	this study	2
Clausiliidae	*Megalauchenia proctostoma forceps* (Loosjes & Loosjes-van Bemmel, 1973)	Loosjes and Loosjes-van Bemmel (1973) [*Tropidauchenia proctostoma forceps*], Vermeulen and Maassen (2003) [*Tropidauchenia proctostoma*], Nordsieck (2011), this study	1, 2
Clausiliidae	*Megalauchenia proctostoma proctostoma* (Mabille, 1889)	Nordsieck (2011), this study	
Clausiliidae	*Oospira abstrusa abstrusa* (Szekeres, 1970)	Szekeres (1970) [*Euphaedusa abstrusa*], Vermeulen and Maassen (2003) [*Euphaedusa* sp. vi-1]^11^, this study	1, 2
Clausiliidae	*Oospira eregia* (Szekeres, 1969)	Vermeulen and Maassen (2003) [*Leptacme* sp. vi-1]^11^, this study	1, 2
Clausiliidae	*Oospira miranda* (Loosjes & Loosjes-van Bemmel, 1973)	Loosjes and Loosjes-van Bemmel (1973) [Formosana (Dextroformosana) miranda], Vermeulen and Maassen (2003) [*Formosana miranda*], Mamos et al. (2021), Sulikowska-Drozd et al. (2022), this study	1, 2
Clausiliidae	*Oospira vanbuensis* (Bavay & Dautzenberg, 1899)	Szekeres (1970) [*Phaedusa umbratica*], Loosjes and Loosjes-van Bemmel (1973) [*Phaedusa paviei*], Vermeulen and Maassen (2003), Sulikowska-Drozd et al. (2018), Mamos et al. (2021), this study	1, 2
Clausiliidae	*Phaedusa dichroa* (Bavay & Dautzenberg, 1899)	Mamos et al. (2021), this study	2
Clausiliidae	*Phaedusa lypra* (Mabille, 1887)	Vermeulen and Maassen (2003) [*Phaedusa backhanensis* (sic)], this study	1, 2
Clausiliidae	*Phaedusa stenothyra* Möllendorff, 1901	Mamos et al. (2021), Dedov et al. (2025), this study	2
Diapheridae	*Sinoennea* sp. vi-3 *sensu* Vermeulen & Maassen 2003	Vermeulen and Maassen (2003)	1
Diapheridae	*Sinoennea* sp. A	this study	2
Dyakiidae	*Quantula* sp. 3 *sensu* Jirapatrasilp et al. 2021	Vermeulen and Maassen (2003) [*Elaphroconcha denserugata*], Jirapatrasilp et al. (2021), this study	1, 2
Enidae	*Apoecus messageri* (Bavay & Dautzenberg, 1900)	Vermeulen and Maassen (2003) [*Mirus tenuistriatus*], this study	1, 2
Euconulidae ^12^	*Liardetia* sp. vi-k2 *sensu* Vermeulen & Maassen 2003^12^	Vermeulen and Maassen (2003)	1
Euconulidae ^12^	*Liardetia* sp. vi-k4 *sensu* Vermeulen & Maassen 2003^12^	Vermeulen and Maassen (2003)	1
Euconulidae ^12^	*Liardetia* sp. vi-o1 *sensu* Vermeulen & Maassen 2003^12^	Vermeulen and Maassen (2003)	1
Euconulidae ^12^	*Liardetia* sp. vi-o3 *sensu* Vermeulen & Maassen 2003^12^	Vermeulen and Maassen (2003)	1
Euconulidae ^12^	*Liardetia* sp. vi-r1 *sensu* Vermeulen & Maassen 2003^12^	Vermeulen and Maassen (2003)	1
Euconulidae ^12^	*Liardetia* sp. vi-r3 *sensu* Vermeulen & Maassen 2003^12^	Vermeulen and Maassen (2003)	1
Helicarionidae	*Chalepotaxis infantilis* (Gredler, 1881)	Vermeulen and Maassen (2003), this study	1, 2
Helicarionidae	*Durgella* sp. A	Vermeulen and Maassen (2003) [*Helicarion* sp. vi-1], this study	1, 2
Helicarionidae	*Durgella* sp. B	Vermeulen and Maassen (2003) [*Helicarion* sp. vi-2]^11^, this study	1, 2
Helicarionidae	*Rahula jucunda* (Bavay & Dautzenberg, 1912)	Vermeulen and Maassen (2003), this study	1, 2
Helicarionidae	*Sesara* sp. vi-01 *sensu* Vermeulen & Maassen 2003	Vermeulen and Maassen (2003), this study	1, 2
Helicarionidae	*Sitala confinis* (Möllendorff, 1901)	Vermeulen and Maassen (2003) [*Liardetia* sp. vi-k3], this study	1, 2
Helicarionidae	*Sitala tricincta* Saurin, 1953^9^	Vermeulen and Maassen (2003) [*Philalanka tricincta*]	1
Helicarionidae	*Teraia contempta* (Bavay & Dautzenberg, 1909)	this study	2
Hypselostomatidae	*Angustopila fabella* Páll-Gergely & Hunyadi, 2015	Vermeulen and Maassen (2003) [*Systenostoma* sp. vi-02 (partly)]^13^, Páll-Gergely et al. (2023)	1
Hypselostomatidae	*Angustopila oostoma* Páll-Gergely & Vermeulen, 2023	Vermeulen and Maassen (2003) [*Systenostoma* sp. vi-02 (partly), *Systenostoma* sp. vi-06]^13^, Páll-Gergely et al. (2023)	1
Hypselostomatidae	*Angustopila vandevenderi* Páll-Gergely & Jochum, 2023	Páll-Gergely et al. (2023)	
Hypselostomatidae	*Aulacospira* sp. vi-02 *sensu* Vermeulen & Maassen 2003	Vermeulen and Maassen (2003)	1
Hypselostomatidae	*Bensonella* sp. A	Vermeulen and Maassen (2003) [*Boysidia* sp. vi-01], this study^10^	1, 2
Hypselostomatidae	*Hypselostoma crossei* Morlet, 1886	Gojšina et al. (2025)	
Hypselostomatidae	*Systenostoma* sp. vi-03 *sensu* Vermeulen & Maassen 2003^14^	Vermeulen and Maassen (2003)	1
Hypselostomatidae	*Systenostoma* sp. vi-07 *sensu* Vermeulen & Maassen 2003^14^	Vermeulen and Maassen (2003)	1
Philomycidae	*Meghimatium pictum* (Stoliczka, 1873)	this study	
Plectopylidae	*Gudeodiscus messageri raheemi* Páll-Gergely & Hunyadi, 2015	Vermeulen and Maassen (2003) [*Plectopylis phlyaria* (partly)], Páll-Gergely et al. (2015b), this study	1, 2
Plectopylidae	*Gudeodiscus phlyarius* (Mabille, 1887)	Vermeulen and Maassen (2003) [*Plectopylis phlyaria* (partly)], Páll-Gergely et al. (2015b), this study	1, 2
Streptaxidae	*Haploptychius diespiter* (Mabille, 1887)	Vermeulen and Maassen (2003), this study	1, 2
Streptaxidae	*Perrottetia daedalea* (Bavay & Dautzenberg, 1909)	Vermeulen and Maassen (2003) [*Perrottetia daedalus* (sic)]	1
Streptaxidae	*Perrottetia* sp. A	this study	2
Trochomorphidae	*Trochomorpha paviei* (Morlet, 1885)	Vermeulen and Maassen (2003) [*Videna paviei*], this study	1, 2
Trochomorphidae	*Trochomorpha saigonensis* (Crosse, 1867)	this study	1, 2
Trochomorphidae	*Trochomorpha* sp. A	Vermeulen and Maassen (2003) [*Videna* sp. vi-1], this study	1, 2
Valloniidae	*Pupisoma dioscoricola* (Adams, 1845)	Vermeulen and Maassen (2003) [*Ptychopatula orcula*], this study	1, 2
Valloniidae	*Pupisoma miccyla* (Benson, 1860)^15^	Vermeulen and Maassen (2003) [*Ptychopatula miccyla*]	1

^1^Based on unpublished data provided by M Yago. ^2^Based on unpublished data provided by F Naggs, DC Raheem and J Ablett. ^3^Based on unpublished data provided by E Ivanova. ^4^Marked with “(?)” in Vermeulen and Maassen (2003). ^5^Taxonomy follows Páll-Gergely et al. (2017b). ^6^Genus uncertain; the *Japonia* species *sensu* Vermeulen and Maassen (2003), which have been examined in the present study, were regarded as members of the genus *Lagocheilus*. ^7^Identification follows Vermeulen and Maassen (2003); the single specimen available to Yamazaki et al. (2007) was presumably subadult. ^8^Correspondence of species based on Yamazaki et al. (2007) and unpublished data provided by JJ Vermeulen. ^9^Taxonomy follows Inkhavilay et al. (2019). ^10^Only material with uncertain identification recorded. ^11^Presumed concordance as no material or images were available to us for comparison. ^12^Genus and family uncertain; the *Liardetia* species *sensu* Vermeulen and Maassen (2003), which have been examined in the present study, were regarded as members of the genera *Kaliella* (Chronidae), *Sitala* (Helicarionidae) and *Microcystina* (Ariophantidae). ^13^Based on Páll-Gergely et al. (2023) and unpublished data provided by JJ Vermeulen. ^14^Note that the name *Systenostoma* has been found to be preoccupied and replaced with *Tonkinospira* (Jochum et al. 2014). ^15^Taxonomy follows Preece et al. (2022).
